# Enhancing salinity tolerance in wheat: the role of synthetic Strigolactone (GR24) in modulating antioxidant enzyme activities, ion channels, and related gene expression in stress responses

**DOI:** 10.1038/s41598-025-08670-3

**Published:** 2025-07-07

**Authors:** Parisa Jariani, Manijeh Sabokdast, Fatemeh Rajabi, Mohammad Reza Naghavi, Beata Dedicova

**Affiliations:** 1 Department of Agronomy and Plant Breeding, College of Agriculture and Natural Resources, University of Tehran, P.O. Box 4111, Karaj, 31587-11167 Iran; 2 Department of Plant Breeding, Swedish University of Agricultural Sciences (SLU), Sundsvägen 10, Alnarp, P.O. Box 190, 234 22 Lomma, Sweden

**Keywords:** Abiotic stress, Antioxidant enzymes, Salinity tolerance, Strigolactones (GR24), *Triticum aestivum* L, Biochemistry, Genetics, Plant sciences

## Abstract

Wheat (*Triticum aestivum* L.) is a vital global crop; however, its productivity is facing increasing threats from soil salinity, which affects a significant portion of arable land worldwide. This study investigates the potential of synthetic Strigolactone (GR24) to enhance salinity tolerance in wheat by examining its effects on antioxidant enzyme activity, ion homeostasis, and gene expression. Three wheat cultivars with varying salinity resistance (Sistan, Pishtaz, and Tajen) were treated with 10 µM GR24 under two salinity levels (5 and 15 dS/m). Salinity stress was applied from the 3–4 leaf stage to tillering. GR24 significantly enhanced the activities of antioxidant enzymes such as ascorbate peroxidase, catalase, and polyphenol oxidase while reducing guaiacol peroxidase activity. Proline content, potassium levels, and concentrations of chlorophyll and carotenoids were markedly increased, while sodium ion accumulation and indicators of oxidative damage (malondialdehyde, hydrogen peroxide, and electrolyte leakage) were reduced. These effects improved leaf water retention and overall stress resilience. Furthermore, polyphenol oxidase activity highlighted a potential novel pathway of Strigolactone action involving interactions with other phytohormones. Gene expression analysis via real-time PCR revealed that GR24 modulates the transcription of stress-responsive genes, including antiporter genes crucial for maintaining Na+/K + homeostasis and reducing ion toxicity. Among the cultivars, Sistan and Tajen exhibited the most robust responses at a salinity level of 15 dS/m. These findings underscore the potential of GR24 as a promising tool for enhancing wheat performance in saline environments.

## Introduction

Wheat (*Triticum aestivum* L.) is one of the most important cereal crops globally, serving as a primary source of calories and protein for a significant portion of the world’s population^1–3^. Its strategic importance in food security and economic stability cannot be overstated. However, wheat productivity is increasingly threatened by soil salinity and other environmental stresses, such as drought and heat stress, which lead to reduced dry weight in leaves, sheaths, and stems, ultimately causing yield reduction^4–7^. This pervasive issue affects approximately 20% of the world’s irrigated lands and is particularly severe in arid and semi-arid regions, including Iran^8^.

Salinity stress disrupts plant water uptake, ion homeostasis, and induces oxidative stress, resulting in reduced growth and yield^9,10^. This major environmental concern constrains plant and crop productivity, affecting over 6% of the world’s land. It negatively influences survival, biomass production, and yield of staple food crops^11^.

To mitigate the harmful effects of salinity stress, wheat plants employ various adaptive mechanisms, including osmoregulation, ion homeostasis, and antioxidant defense systems^12^. Additionally, management strategies such as arbuscular mycorrhizal fungi application, plant growth-promoting rhizobacteria, exogenous phytohormone treatments, seed priming, and nutrient management have shown promising improvements in wheat performance under saline conditions^13,14^. In addition to salinity, drought stress is another major environmental factor that severely impacts wheat growth, leading to reduced photosynthesis and respiration efficiency, ultimately lowering yield^4,15^. To cope with water deficits, wheat plants activate physiological and ecological mechanisms, including enhanced root development, modified growth rates, and improved water use efficiency^16^. Key drought adaptation strategies include biomass allocation adjustments, stomatal regulation to minimize transpiration loss, and maintenance of relative water content, though severe drought still hinders nutrient uptake, further exacerbating yield declines^17–20^.

Heat shock transcription factors (Hsfs) play a crucial regulatory role in how wheat responds to heat stress. One such factor, *TaHsfA2-1*, was cloned from wheat and found to encode a 346-amino acid residue protein. This protein, localized in the nucleus under normal conditions, is expressed in nearly all wheat tissues, with the highest levels in mature leaves. The expression level of *TaHsfA2-1* can be enhanced by heat stress, PEG-induced osmotic stress, and signaling molecules such as H₂O₂ and SA^21^.

Different methods of manganese (Mn) application significantly impact wheat productivity, grain biofortification, and economic returns under conventional tillage (CT) and no-tillage (NT) systems. Research has shown that Mn nutrition, regardless of application method, enhances wheat grain yield. Specifically, osmopriming (seed priming with a 0.1-M Mn solution) and foliar application (0.25-M Mn solution) have been particularly effective in boosting yield and yield components, with foliar Mn application leading to the highest grain Mn concentration^22^.

Environmental stresses—including salinity, drought, and heat stress—pose significant challenges to wheat productivity by disrupting growth, development, grain yield, and quality. Wheat plants activate multiple physiological, biochemical, and molecular mechanisms to counteract stress effects at cellular, tissue, and whole-plant levels. Adaptation and management strategies—including molecular breeding, genetic engineering, CRISPR-Cas gene editing, nano-based agricultural technologies, and optimized agronomic practices—hold promising potential to mitigate stress effects and maximize wheat production and nutritional quality^23^.

Strigolactones (SLs), a class of carotenoid-derived plant hormones, play a crucial role in regulating developmental processes such as shoot branching, root architecture, and symbiotic interactions with mycorrhizal fungi^24–27^. Beyond their developmental functions, SLs have gained significant attention for their ability to enhance plant resilience to abiotic stresses, particularly drought and salinity^28,29^. However, despite extensive research on SLs, critical gaps remain in understanding their cultivar-specific responses, long-term effects, and the precise gene regulation pathways governing stress adaptation.

The synthetic analog GR24 has been widely used to investigate the molecular and physiological mechanisms by which SLs contribute to stress tolerance^30^. GR24 is known to modulate antioxidant enzyme activities, mitigating oxidative damage induced by ROS through enzymes such as ascorbate peroxidase APX, CAT, and PPO^31–36^. Moreover, SLs influence the expression of genes critical for ion homeostasis and transport, including *TaAKT2*, *TaHAK*, *TaSOS1*, *NHX*, *TaP5CS*, and *HKT1*—key players in maintaining osmotic balance and enhancing salinity tolerance. Despite these insights, a deeper understanding of how SLs interact with specific genotypes under prolonged stress conditions remains largely unexplored.

This study examines the effects of GR24 on the salinity tolerance of three wheat cultivars—Sistan, Pishtaz, and Tajen—each exhibiting different resistance levels. By assessing physiological and biochemical responses, including antioxidant enzyme activities, proline accumulation, ion content, and chlorophyll levels, this research aims to elucidate the mechanisms underlying SL-mediated stress adaptation. Addressing the gaps in cultivar-specific responses and gene regulation will provide critical insights for the application of SLs in enhancing crop resilience in saline environments, contributing to sustainable agriculture and food security.

Previous studies have established the fundamental role of SLs in plant stress physiology, demonstrating their influence on drought and salinity tolerance through hormonal crosstalk and stress-responsive gene expression^37^. However, unanswered questions regarding their long-term impacts and genotype-dependent effects persist. Building on existing knowledge, this study focuses on the practical application of GR24 in wheat under salinity stress, integrating molecular, biochemical, and physiological analyses to advance SL-based agricultural innovations^38^.

## Results

### Impact of salinity and Strigolactone on RWC in different cultivars

**Fig. 1:**
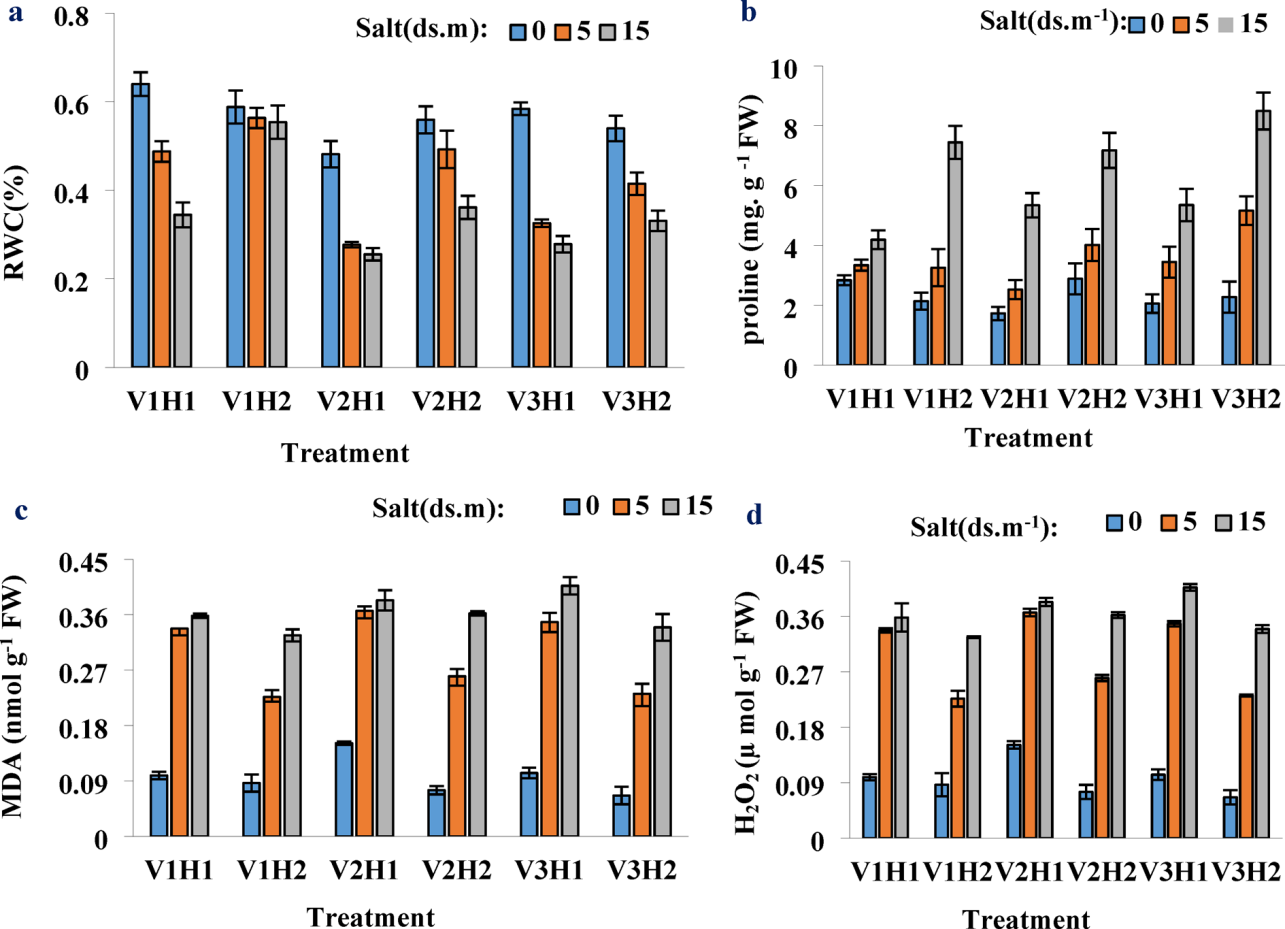
The effects of different salinity levels (0, 5, and 15 dS/m) on various physiological and biochemical parameters in plants under different treatments (V1H1, V1H2, V2H1, V2H2, V3H1, and V3H2). (**A**) Relative water content (RWC), (**B**) Proline content, (**C**) lipid peroxidation (MDA) levels, and (**D**) Hydrogen peroxide (H_2_O_2_) concentration. Treatments are as follows: V1: Sistani, V2: Pishtaz, V3: Tajan, H1: without hormone (GR24 at 0 µM), H2: with hormone (GR24 at 10 µM).

The study investigated the effects of salinity and strigolactone on the relative water content (RWC) in leaves of various cultivars. Significant interactions between salinity and strigolactone were observed, with notable effects at 1% for both cultivar*strigolactone and salinity*strigolactone interactions and at 5% for salinity*cultivar interactions. V1 - Sistani, recognized for its resilience, displayed the highest RWC (61%) regardless of hormone treatment, showing no significant difference under non-stress conditions. This demonstrates that Sistani maintains optimal water retention even in challenging environments. V2 - Pishtaz exhibited the most substantial decrease in RWC (25%) under non-hormone conditions, identifying it as the most sensitive cultivar. However, strigolactone treatment boosted its RWC by 11%, suggesting its potential role in enhancing drought tolerance. V3 - Tajan - It is implied that strigolactone treatment generally improved leaf water retention compared to non-hormone conditions across all cultivars. This indicates a consistent positive effect of strigolactone on plant hydration. Strigolactone treatment (H2) enhanced leaf water retention in all cultivars compared to non-hormone conditions (H1). Sistani showed the highest change in RWC (21%) between hormone-treated and untreated conditions under 15 dS/m salinity, reinforcing its adaptability under stress conditions. These findings underscore the significance of RWC as a marker of plant water status, influenced by osmotic stress and ion accumulation (Fig. 1a).

### Proline content

Proline content was significant for the interaction effect of salinity*Strigolactone at the 1% level and the impact of cultivar*salinity at the 5% level, while the interaction effects of cultivar*salinity*Strigolactone and cultivar*Strigolactone were not significant. The highest increase in proline content was observed in the sensitive Tajan cultivar under 15 dS/m salinity, both with and without hormone application, at 41%. This suggests that a factor in this cultivar may enhance the expression of proline-encoding genes, making it particularly reactive to stress conditions. The resistant Sistani cultivar showed the lowest proline accumulation under non-hormonal conditions. GR24 application at zero and 5 dS/m salinity slightly reduced proline levels in Sistani, likely due to its high resistance and the ability to mitigate minor damages through other means or sufficient endogenous Strigolactone presence. This supports the notion that highly resistant cultivars rely on alternative defense mechanisms against stress. The sudden increase in proline under non-hormonal conditions in the Pishtaz cultivar also indicates its relative resistance (Fig. 1b).

### Lipid membrane peroxidation (MDA)

In this experiment, the interaction effect of salinity*Strigolactone on MDA was significant at the 5% level, while the impact of cultivar*Strigolactone, cultivar*salinity, and cultivar*salinity*Strigolactone was not significant. The resistant Sistani cultivar showed the least changes in MDA levels under both salinity and hormone application conditions, as well as under non-hormonal conditions. This highlights its ability to maintain cellular integrity under stress. The minimal lipid peroxidation in Sistani suggests a well-developed antioxidant defense system. In contrast, the Tajan cultivar exhibited the best response to hormone application, with a 27% reduction in MDA levels, demonstrating the potential of Strigolactone in reducing oxidative damage. The enhanced protection against oxidative stress in hormone-treated plants supports its application in regions prone to salinity. The Pishtaz cultivars also showed significant reductions in MDA with hormone application, though less than Tajan, indicating that hormone treatment is beneficial in lowering lipid peroxidation. The results suggest that Strigolactone contributes to stabilizing membrane integrity in stressed plants (Fig. 1c).

### Hydrogen peroxide (H_2_O_2_)

In this experiment, the interaction effects of cultivar*Strigolactone, cultivar*salinity, cultivar*salinity*Strigolactone, and salinity*Strigolactone on H_2_O_2_ levels were significant at the 1% level. The minimal changes in H_2_O_2_ levels with hormone application compared to non-hormonal conditions are likely due to the neutralization of this reactive oxygen species by other antioxidant mechanisms. This suggests that wheat plants possess intrinsic regulatory processes to manage oxidative stress effectively. The presence of endogenous Strigolactone likely plays a role in modulating ROS under stress conditions. Additionally, the application of GR24 may reduce the plant’s need for endogenous Strigolactone production, further influencing stress responses. The observed variation in H_2_O_2_ levels highlights the complexity of oxidative stress mitigation pathways in wheat cultivars. Another reason could be the high presence of endogenous Strigolactone in wheat, resulting in less pronounced changes with GR24 application, pointing to the plant’s ability to adjust hormone levels naturally (Fig. 1d).

### Chlorophyll a content

The interaction effects of cultivar*salinity*Strigolactone, cultivar*salinity, and cultivar*Strigolactone were significant at the 1% level, whereas the interaction effect of salinity*Strigolactone was not significant. These results suggest that genetic differences play a crucial role in determining chlorophyll stability under salinity stress. The highest chlorophyll content was observed in the resistant cultivar Sistan, with a notable 12% increase at a salinity level of 15 dS/m. This increase highlights Sistan’s ability to maintain efficient photosynthesis despite high salinity stress. On the other hand, the cultivar Tajen exhibited the lowest chlorophyll content, showing minimal reduction under 15 dS/m salinity without hormone application. This suggests that Tajen’s inherent sensitivity to salinity limits its photosynthetic capacity.

For Tajen, hormone application only slightly mitigated changes without altering baseline chlorophyll levels under non-stress conditions. This suggests that Strigolactone treatment alone is insufficient to boost chlorophyll production in Tajen significantly.

**Fig. 2:**
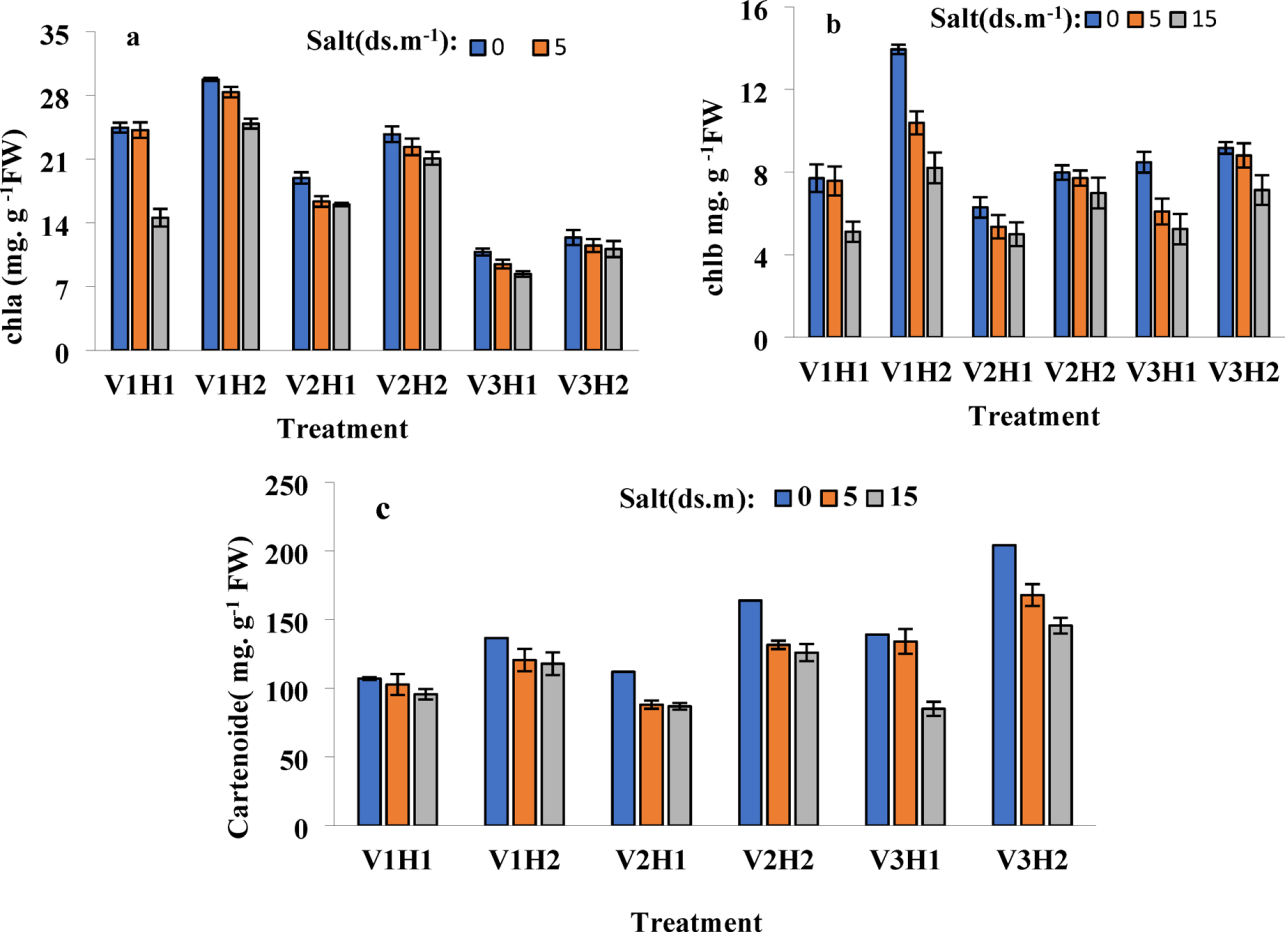
Different salinity levels (0, 5, and 15 dS/m) affect various physiological and biochemical parameters in plants under different treatments. The parameters analyzed include (**a**) chlorophyll a (Chla), (**b**) chlorophyll b (Chlb), and (**c**) carotenoid levels. Treatments are as follows: V1: Sistani, V2: Pishtaz, V3: Tajan, H1: without hormone (GR24 at 0 µM), H2: with hormone (GR24 at 10 µM).

The cultivars Pishtaz and Sistan demonstrated intermediate responses, with hormone application increasing initial chlorophyll levels under both stress and non-stress conditions and moderating the slope of changes, indicating high responsiveness to Strigolactone (Fig. 2a). This responsiveness suggests that hormonal regulation plays a key role in adjusting photosynthetic performance in these cultivars.

### Chlorophyll b content

Significant interaction effects were noted for cultivar*Strigolactone and cultivar*salinity at the 1% level, and for cultivar*salinity*Strigolactone at the 5% level, while the salinity*Strigolactone interaction was not significant. These findings suggest that the combined effect of genotype and stress conditions has a greater impact than hormonal treatment alone. The resistant cultivar Sistan had the highest chlorophyll b content under non-stress conditions and exhibited the most substantial increase in chlorophyll b levels. This suggests that Sistan can effectively regulate its photosynthetic pigments in response to changing environmental conditions. In contrast, other cultivars showed negligible changes in chlorophyll b under both stress and non-stress conditions, regardless of hormone application (Fig. 2b). This minimal variation implies that chlorophyll b is less responsive to Strigolactone treatments compared to chlorophyll a.

### Carotenoid content

Interaction effects for salinity*Strigolactone, cultivar*Strigolactone, and cultivar*salinity were significant at the 1% level, while the interaction effect of cultivar*salinity*Strigolactone was significant at the 5% level. These considerable interactions highlight the complex role of environmental and hormonal factors in regulating carotenoid accumulation. The sensitive cultivar Tajen recorded the highest carotenoid content, with GR24 application significantly enhancing carotenoid levels across all salinity treatments. This suggests that Strigolactone plays a key role in modulating stress-induced pigment biosynthesis in sensitive cultivars. Although hormone application increased carotenoid levels in other cultivars, these changes were insignificant. This indicates that the natural carotenoid regulation in resistant cultivars may be less dependent on hormonal treatments.

The increase in carotenoids may be due to GR24 reducing the plant’s internal Strigolactone synthesis needs, enhancing osmotic potential and water retention. This highlights the potential cross-talk between Strigolactone and osmotic balance mechanisms in plants.

Additionally, GR24’s interaction with internal Strigolactone could amplify ethylene signaling, thereby increasing carotenoid levels (Fig. 2c). These findings suggest that hormonal treatments influence multiple biochemical pathways that contribute to stress adaptation.

### Total chlorophyll content

Significant interaction effects were identified for salinity*Strigolactone, a cultivar*salinity*Strigolactone at the 5% level, and cultivar*salinity at the 1% level, with no significant effect for cultivar*Strigolactone. These results indicate that salinity and genotype have a greater impact on total chlorophyll content than Strigolactone alone. In the Sistan cultivar, total chlorophyll content decreased under 5 dS/m salinity with hormone application but increased under 15 dS/m salinity, likely due to the cultivar’s inherent resistance to salinity. This suggests that moderate salinity levels may temporarily affect photosynthesis, but high salinity triggers adaptive responses.

Conversely, the Pishtaz cultivar showed an increase in total chlorophyll under 5 dS/m salinity with hormone application but a decrease under 15 dS/m salinity, indicating the ineffectiveness of the treatment for this trait in Pishtaz. These patterns suggest that Pishtaz may require additional protective mechanisms to sustain chlorophyll stability under extreme stress.

The Tajen cultivar demonstrated an increase in total chlorophyll across all salinity levels. This unexpected.

The trend suggests that Tajen may activate compensatory mechanisms to maintain photosynthetic activity despite its overall sensitivity to salinity.

### Electrolyte leakage (ELI)

In this experiment, the interaction effects of cultivar*Strigolactone, cultivar*salinity, and cultivar*salinity*Strigolactone on electrolyte leakage were significant at the 1% level, while the impact of salinity*Strigolactone was not significant. This suggests that cultivar-specific responses and the combined effect of salinity and hormone treatments are more important in determining membrane stability.

The reduction in electrolyte leakage was more pronounced in the Tajen cultivar compared to other cultivars, which can be attributed to its sensitivity. This suggests that Tajen’s membrane integrity is more affected by stress, making it a suitable candidate for studying stress susceptibility mechanisms. Under non-hormonal conditions, this cultivar exhibited a 10% increase in electrolyte leakage, which decreased to 4% with foliar application. The hormonal treatment appears to enhance membrane protection, potentially through antioxidant defense mechanisms. In Tajen, hormone application reduced leakage under both stress and normal conditions. This consistent reduction suggests that Strigolactone plays a stabilizing role in maintaining membrane integrity, regardless of the severity of stress (Table 1).

**Table 1:** Primer sequences of Stress-Responsive genes in *T. Aestivum.*

Accession number	Gene name	Product length	Annealing temp	Sequence
KJ563229.1	*TaSOS1*-F	148	61	F: CTGAAGACAGAGAACCGAAC
	*TaSOS1*-R		60	R: CTCTAGCCTCCACCTGATAA
OM200013.1	*TaNHX1*-F	150	61	F: GGTTCACCCATAGAGAGGAG
	*TaNHX1*-R		60	R: CCCAAGAGCAAAACTGAGTG
XM_044553598.1	*TaAKT2*-F	145	60	F: CCGATCACCATCTACGACT
	*TaAKT2*-R		60	R: GGTTTCTCACGAACGGATG
U16709.1	*TaHKT1*-F	146	61	F: GCAACACCCAATGGAGATAC
	*TaHKT1*-R		61	R: CAGTGATGCAGGCAACTATC
JF495466.1	*TaHAK*-F	140	60	F: CTGATCTACCTCTCGTCCATAC
	*TaHAK*-R		59	R: CTCGTACCAGTACCTCTTCAC
KC175596.1	*TaP5CS*-F	150	60	F: CCAGGAAAGATAGCAAGCC
	*TaP5CS*-R		59	R: AAACCATCAGCAACCTCTG
AB181991.1	*TaActin*-F	130	59	F: CACGCTTCCTCATGCTATC
	*TaActin*-R		59	R: CTGACAATTTCCCGCTCAG

The least changes were observed in the Sistan cultivar, which has a more stable membrane due to its salinity resistance. This highlights the genetic advantage of Sistan in maintaining ion balance under adverse conditions. Alongside Sistan, the Pishtaz cultivar also showed minimal changes. The similarity between these cultivars in electrolyte leakage patterns indicates a shared physiological mechanism for salinity resistance.

The analysis of variance for EL, RWC, MDA, Proline, H₂O₂, Chla, Chlb, and Carotenoid under salt stress and Strigolactone hormone spraying in wheat cultivars is presented in Table 2. This dataset provides valuable insights into the relationship between oxidative stress markers and membrane stability under different treatments.

**Table 2:** ANOVA for mean squares of physiological and biochemical parameters in *T. aestivum* *p < 0.05, **p < 0.01, ns = not significant; electrolyte leakage (EL), lipid peroxidation (MDA) levels, hydrogen peroxide (H_2_O_2_) concentration, relative water content (RWC), proline content, chlorophyll a (Chla), chlorophyll b (Chlb) and carotenoid levels.

Mean of Squares									
(S.O.V)	df	ELI	MDA	H_2_O_2_	Proline	RWC	Chla	Chlb	Carotenoid
Cultivar (CV)	2	0.02678233*	0.00240420*	0.00006527*	6.799*	0.067*	586.383*	20.333*	3744.952*
Hormone(H)	1	0.14141486*	0.06414168*	0.00063427*	31.454*	0.074*	520.751*	77.696*	19076.416*
Salt(s)	2	1.20880809*	0.44255268*	0.00010710*	80.284*	0.2*	109.436*	35.578*	8718.624*
H×S	2	0.00921720*	0.00911551**	0.00000057^**ns**^	8.576*	0.024*	1.689ns	1.475ns	282.430*
V×H	2	0.00392396*	0.00084516^**ns**^	0.00006121*	0.680^**ns**^	0.013*	21.765*	6.3825*	1682.92*
V×S	4	0.00444622*	0.00059575^**ns**^	0.00000447*	2.172**	0.008**	14.179*	3.175*	946.422*
V×S×H	4	0.00300054*	0.00059711^**ns**^	0.00000458*	0.964^**ns**^	0.005ns	11.492*	2.604**	116.632**
Error		0.00011220	0.00072226	0.00000035	1.089	0.003	2.332	0.890	26.398
CV (%)		7.71	10.69	8.02	27.1	12.22	8.53	12.96	4.12

### Sodium (Na^+^) and potassium (K^+^) content

**Fig. 3:**
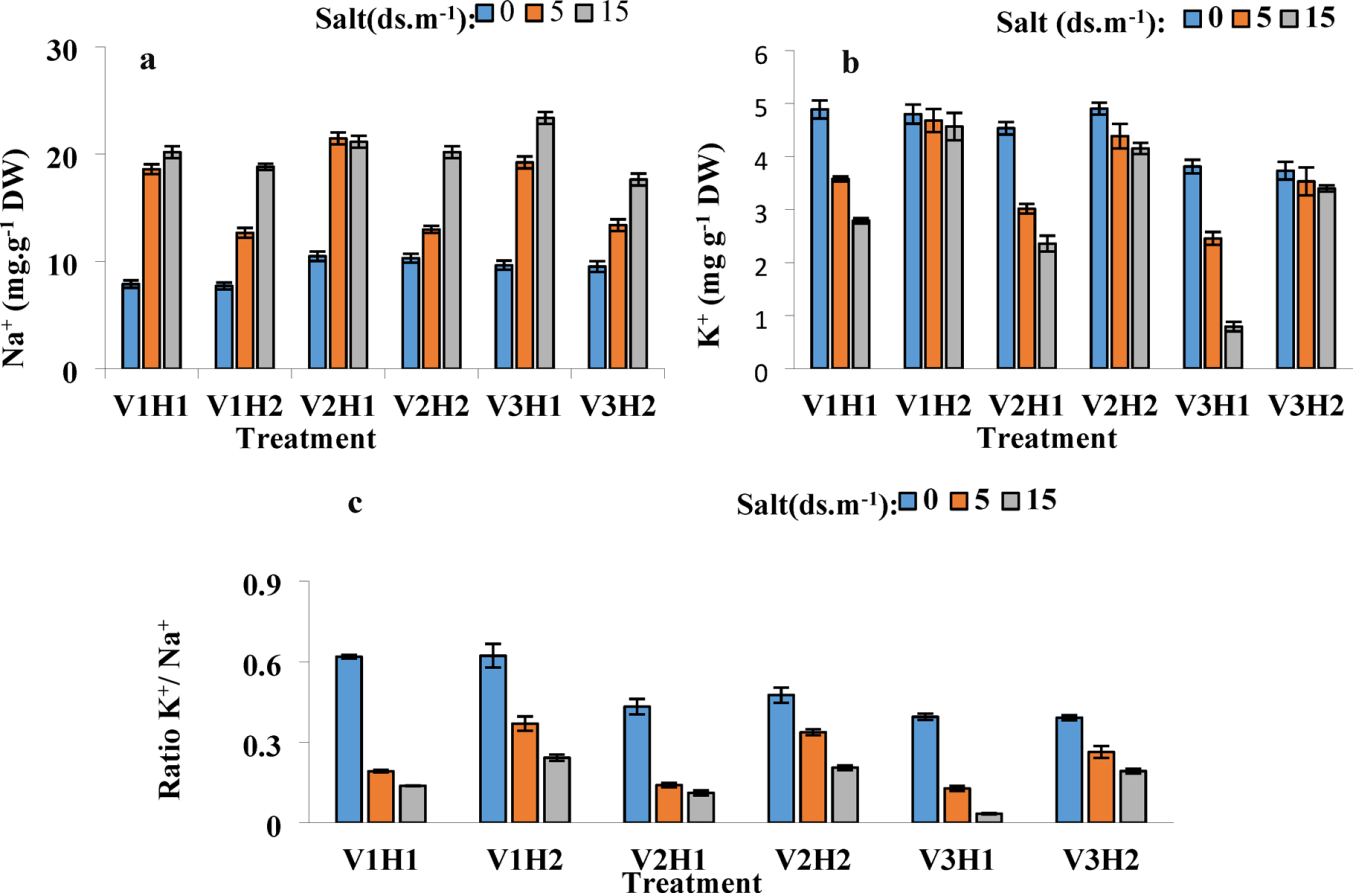
Effects of GR24 Foliar Application on Ion Content and Ratio in Wheat Cultivars Under Salinity Stress (0, 5, and 15 dS/m). The figure illustrates the effects of GR24 foliar application on sodium content (Na⁺), potassium content (K⁺), and the potassium-to-sodium ratio (K⁺/Na⁺) in wheat cultivars. Treatments are as follows: V1: Sistani, V2: Pishtaz, V3: Tajan, H1: without hormone (GR24 at 0 µM), H2: with hormone (GR24 at 10 µM).

In this experiment, the interaction effects of salinity*Strigolactone, cultivar*salinity*Strigolactone,

cultivar*salinity, and cultivar*Strigolactone on sodium content were significant at the 1% probability level. This highlights the intricate regulatory dynamics governing sodium accumulation in response to environmental and hormonal stimuli. The GR24 treatment significantly reduced sodium content in the leaves of the sensitive Tajen cultivar. This suggests that hormonal application enhances sodium extrusion mechanisms, providing a protective effect against salt-induced toxicity. However, it had a lesser impact on the Sistan and Pishtaz cultivars, likely due to their inherent resistance to the disease. Their ability to naturally regulate sodium uptake indicates strong genetic control over ion transport mechanisms. The reduction in sodium content in wheat leaves may be attributed to the positive effect of GR24 on sodium transporters and their expulsion. This suggests that hormonal signaling influences the expression of transporter genes, facilitating the active removal of excess sodium. For potassium content, the interaction effects of salinity*Strigolactone, cultivar*salinity*Strigolactone, cultivar*salinity, and cultivar*Strigolactone were significant at the 1% probability level. These considerable interactions highlight the crucial role of potassium regulation in maintaining ionic homeostasis. Foliar application of GR24 significantly increased potassium content in all cultivars. This increase suggests that strigolactone enhances potassium uptake, thereby reinforcing its protective role in cellular function. The highest growth was observed in the Tajen cultivar, where potassium content increased by 60% under a 15 dS/m salinity level. This suggests a strong hormonal effect in potassium retention, further supporting the idea that Tajen relies on external signaling for ion balance. However, this increasing trend was not observed under non-stress conditions with hormone application. The absence of a significant effect under normal conditions indicates that potassium homeostasis is predominantly stress-dependent. Therefore, it can be concluded that the presence of sodium itself may enhance the synthesis of endogenous Strigolactone, acting as an environmental signal for Strigolactone production. This feedback mechanism suggests that plants dynamically regulate hormone synthesis in response to ionic stress levels. This, in turn, strengthens sodium transporters, facilitating sodium expulsion and potassium uptake under high salinity conditions (Fig. 3a and b). These findings contribute to our understanding of how hormonal treatments interact with ion transport processes to enhance stress resilience.

### Potassium to sodium ratio (K^+^/Na^+^)

The interaction effects of salinity*Strigolactone, cultivar*salinity*Strigolactone, and cultivar*salinity on the potassium to sodium ratio were significant at the 1% probability level, while the impact of cultivar*Strigolactone was not significant. This suggests that salinity stress plays a more dominant role in K⁺/Na⁺ balance than hormonal treatment alone. The potassium-to-sodium ratio significantly decreased under salinity stress without hormone application, but was markedly improved with GR24 foliar application. This highlights the ability of hormonal treatment to restore ionic balance in stressed conditions. The highest K⁺/Na⁺ ratio was observed in the Sistan cultivar, which is notable given its resistance to salinity. This finding reinforces the notion that Sistan possesses superior ion regulation mechanisms, which contribute to its robust tolerance to salinity. Since sodium uptake interferes with potassium homeostasis, maintaining a balanced cytosolic K⁺/Na⁺ ratio is crucial for salinity tolerance. A disrupted balance can lead to metabolic stress, which in turn affects overall plant growth and functionality. Achieving this homeostatic balance requires the activity of Na⁺ and K⁺ channels (Fig. 3c). The efficient regulation of these channels plays a crucial role in mitigating ion toxicity under high-salinity conditions. Analysis of variance of Na⁺, K⁺, and Na⁺/K⁺ ratio under salt stress and Strigolactone hormone spraying in wheat cultivars is presented in Table 3. This statistical analysis offers a valuable reference for further studies on ion transport mechanisms in wheat genotypes.

**Table 3:** ANOVA results for sodium (Na⁺), potassium (K⁺), and potassium-to-sodium ratio (K⁺/Na⁺) in wheat cultivars subjected to different levels of salinity stress and Strigolactone hormone treatments. (S.O.V) refers to sources of variation, and Df represents degrees of freedom. Significance levels are indicated as *p < 0.05 (statistically significant), **p < 0.01 (highly significant), and Ns (not significant).

(S.O.V)	df	Na^+^	K^+^	Na^+^/K^+^ ratio
Cultivar (CV)	2	8.224*	6.131*	0.021*
Hormone(H)	1	81.956*	17.597*	0.084*
Salt(s)	2	75.512*	13.261*	0.084*
H×S	2	14.042*	4.764*	0.0168*
V×H	2	5.231*	0.281*	0.00021^ns^
V×S	4	3.046*	0.124*	0.000791*
V×S×H	4	4.290*	0.185*	0.000896*
Error		0.448	0.00316	0.000129
CV (%)		3.63	5.6	7.01

### Protein content

The total protein content was significant only for cultivar at the 1% level and for hormone at the 5% level, while other effects were not significant. This indicates that genetic differences among cultivars and hormonal treatments have a more pronounced influence on protein content than salinity alone. Overall, total protein content decreased with increasing salinity under non-hormonal conditions. However, this decrease was not substantial, and a slight increase was observed at 5 dS/m salinity. This suggests that wheat cultivars exhibit some level of protein stability under moderate salinity, minimizing the detrimental impact of salt stress. Under hormone application and 5 dS/m salinity, the Sistan cultivar at zero salinity showed an increase in protein content. This response implies that GR24 treatment may help maintain or enhance protein synthesis, contributing to stress resilience.

The lowest protein content was observed in the Tajen cultivar, and the application of hormones further exacerbated this decrease. This reduction indicates that the sensitivity of the Tajen cultivar to stress may extend beyond salinity, potentially affecting protein metabolism under hormonal influence. It appears that changes in protein content are closely related to stress intensity and do not vary significantly unless the stress level becomes dangerously high. This highlights the importance of threshold effects, where stress must exceed a critical level to induce significant alterations in protein levels. Additionally, since wheat is relatively salt-tolerant, total protein content does not exhibit substantial changes. However, some of the measured proteins may have lost their proper structure and activity due to NaCl acting as a denaturing agent. This reinforces the idea that while total protein content remains stable, functional impairments at the molecular level could still occur due to prolonged exposure to salinity.

### Ascorbate peroxidase (APX) activity

**Fig. 4:**
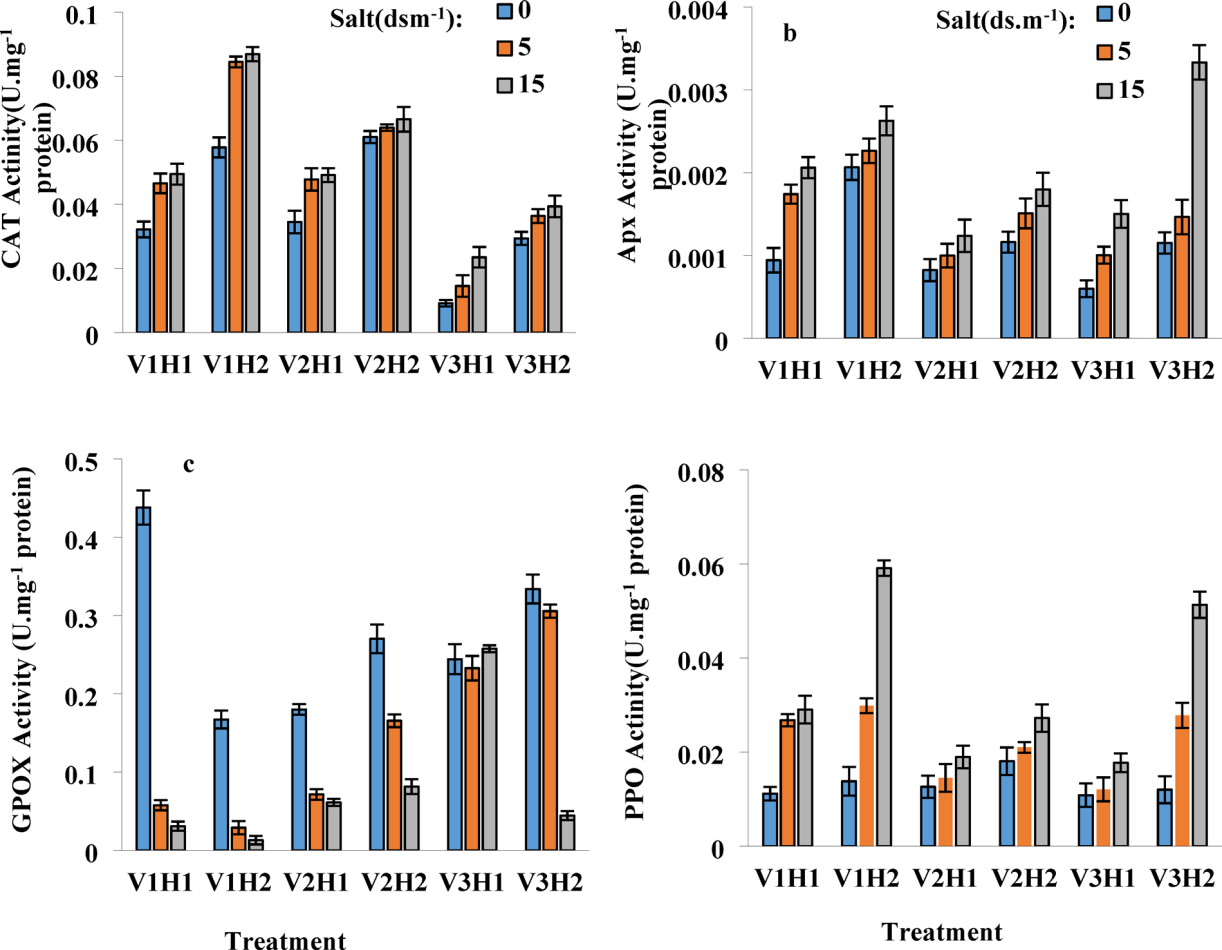
Effect of GR24 on Activity of Antioxidant Enzymes in Wheat Cultivars Under Salinity Stress (0, 5, and 15 dS/m NaCl). (**A**) CAT activity (Catalase) (U mg^− 1^ Protein), (**B**) APX activity (ascorbate peroxidase) (U mg^− 1^ Protein), (**C**) GPOX activity (Guaiacol peroxidase (U mg^− 1^ Protein), (**D**) PPO activity (Polyphenol Oxidase) (U mg^− 1^ Protein), and Treatments are as follows: V1: Sistani, V2: Pishtaz, V3: Tajan, H1: without hormone (GR24 at 0 µM), H2: with hormone (GR24 at 10 µM).

The activity of ascorbate peroxidase was significant for the interaction effect of salinity and Strigolactone at the 5% level, as well as for the impact of cultivar salinity, Strigolactone, and cultivar Strigolactone at the 1% level. These results suggest that both salinity and hormonal treatments play a critical role in modulating antioxidant enzyme activity across different wheat cultivars. The use of GR24 significantly increased ascorbate peroxidase activity even at zero salinity, with the highest activity observed at 15 dS/m salinity across all cultivars. This indicates that Strigolactone enhances the plant’s enzymatic antioxidant defense, potentially reducing oxidative stress under saline conditions. The Tajen cultivar showed the most significant increase in APX activity with foliar application, while the Pishtaz cultivar exhibited the least responsiveness. This disparity suggests genotypic differences in APX regulation, indicating that some cultivars are more reactive to hormone-induced stress mitigation. The lowest APX activity was observed in all cultivars at zero salinity without foliar application, confirming that stress-inducing factors are necessary to trigger enhanced antioxidant enzyme activity in wheat cultivars. Changes in APX activity in the Sistan cultivar were more gradual compared to other cultivars (Fig. 4b), indicating that Sistan exhibits a more stable and controlled response to oxidative stress, which may contribute to its greater resilience.

### Guaiacol peroxidase (GPOX) activity

The activity of GPOX was significant for the interaction effects of salinity, Strigolactone, cultivar salinity, Strigolactone, cultivar salinity, and cultivar Strigolactone at the 1% level. These findings indicate that multiple factors influence peroxidase activity, highlighting the complex regulatory mechanisms involved in stress adaptation.

In this experiment, GPOX activity decreased in the Sistan and Tajen cultivars at 5 dS/m salinity, both with and without hormone application; however, it increased at 15 dS/m salinity. This suggests that peroxidase activity may be strongly induced under higher salinity stress, playing a vital role in cellular defense mechanisms. In the Pishtaz cultivar, the trend was entirely downward. The consistent decrease in GPOX activity may indicate that this cultivar has a weaker antioxidant response to salinity compared to Sistan and Tajen. GR24 treatment increased GPOX activity at zero salinity in the Tajen and Pishtaz cultivars, while a decrease was noted in the Sistan cultivar. This highlights the differential effects of Strigolactone among genotypes, where some cultivars benefit more from hormone applications than others. Overall, GR24 was ineffective in the resistant Sistan cultivar, likely due to its inherent resistance and the neutralization of reactive oxygen species through other mechanisms, as the highest catalase activity was observed in this cultivar. This implies that Sistan relies on alternative antioxidant pathways, reducing its dependence on GPOX under stress conditions. Conversely, foliar application in the Pishtaz cultivar increased GPOX activity at all concentrations (Fig. 4c). This suggests that Pishtaz benefits from external hormonal stimulation to activate its oxidative defense system.

### Catalase (CAT) activity

Catalase activity was significant for the effects of cultivar*salinity*Strigolactone, cultivar*salinity, and cultivar*Strigolactone at the 1% level, but not for the interaction effect of salinity*Strigolactone. This suggests that cultivar-specific responses and salinity have a greater influence on catalase activity than hormonal treatments alone. The highest increase in catalase activity following hormone application was observed in the Sistan cultivar, resulting in a 50% increase in catalase activity. This suggests that Sistan possesses an efficient oxidative defense mechanism that benefits from hormonal supplementation. The lowest catalase activity, both with and without hormone application, was in the sensitive Tajen cultivar. This reinforces the notion that Tajen struggles with oxidative stress management, making it more vulnerable to salinity-induced damage. The Pishtaz cultivar exhibited behavior similar to the Sistan cultivar under non-hormonal conditions, indicating its resistance in terms of catalase activity. This highlights its ability to maintain oxidative balance without external hormonal support. Overall, changes in catalase activity in the Pishtaz cultivar were very mild after hormone application (Fig. 4a). This suggests that Pishtaz does not rely heavily on strigolactone for stress mitigation, unlike other cultivars.

### Polyphenol oxidase (PPO) activity

Polyphenol oxidase (PPO) activity was significantly influenced by the interaction effects of salinity*Strigolactone, cultivar*salinity*Strigolactone, cultivar*salinity, and cultivar*Strigolactone at the 1% level. These interactions suggest that both salinity and hormone treatments play a significant role in determining enzymatic activity across various wheat cultivars.

Across all cultivars, PPO activity increased under non-hormonal conditions, with GR24 treatment further enhancing this activity. This trend suggests that Strigolactone application amplifies stress-induced defense mechanisms, potentially improving structural integrity and resistance against environmental challenges.

This rise in PPO activity could be attributed to the sampling period during the wheat tillering stage, which involves the formation of the main stem and requires the production of woody and secondary tissues. As wheat transitions through this developmental phase, the increased PPO activity may contribute to strengthening plant tissues against oxidative and pathogen-related stressors.

Strigolactone hormones contribute to the thickening and formation of these secondary tissues, protecting against pathogens, possibly by modulating polyphenol oxidase (PPO) activity. This further supports the idea that hormonal regulation influences biochemical defense responses, reinforcing the plant’s natural resistance strategies. In the resistant Sistan cultivar, PPO activity exhibited a significant increase even without hormone application, whereas in other cultivars, the increase was more gradual. This suggests that certain genetic traits in Sistan may predispose it to stronger innate defense mechanisms, giving it a comparative advantage under stress conditions.

The generation of oxidative signals could also be a contributing factor to the observed increase in PPO activity, thereby enhancing the plant’s resistance mechanisms. These oxidative responses may serve as signaling cues to activate additional protective pathways, ensuring the plant remains resilient under varying environmental conditions.

These results highlight the role of Strigolactone and salinity in modulating PPO activity across different cultivars, with significant implications for enhancing plant resistance to environmental stresses and pathogens (Fig. 4d) (Table 4). This insight could be valuable for crop breeding programs aimed at developing varieties with superior stress tolerance and disease resistance.

**Table 4:** ANOVA for CAT (Catalase), APX (ascorbate peroxidase), GPOX (Guaiacol peroxidase) and PPO (Polyphenol Oxidase) activities in wheat cultivars( Sistani, Pishtaz, and Tajan), under Salt stress (0, 5, and 15 dS/m NaCl) and Strigolactone treatment (0,10 µM), (*p < 0.05, **p < 0.01, ns = not significant).

(S.O.V)	df	Protein	CAT	APX	PPO	GPX
Cultivar (CV)	2	1.516*	0.004032*	0.000002*	0.000427*	0.0719*
Hormone(H)	1	5.955**	0.012508*	0.000012*	0.001422*	0.046*
Salt(s)	2	1.589^ns^	0.001407*	0.000005*	0.002485*	0.192*
H×S	2	0.184^ns^	0.000037^ns^	0.000002**	0.000875*	0.021*
V×H	2	0.169^ns^	0.000263*	0.000003*	0.000178*	0.025*
V×S	4	0.182^ns^	0.000085*	0.000003*	0.000200*	0.018*
V×S×H	4	0.327^ns^	0.000095*	0.000006*	0.000114*	0.010*
Error		0.626	0.00002111	0.00000010	0.0000199	0.000087
CV (%)		4.7	9.99	19.39	21.7	6.37

### Correlation analysis

The analysis revealed strong interconnections among various physiological and biochemical factors. Chlorophyll a (Chl a) and b (Chl b) were positively correlated with carotenoids, relative water content (RWC), K, and the potassium-to-sodium ratio (K/Na), indicating a synergistic relationship between photosynthetic pigments and ionic balance. Additionally, Chla demonstrated significant correlations with catalase activity, while ELI strongly correlated with Na, MDA, and H₂O₂, reinforcing the role of oxidative stress in determining salinity tolerance mechanisms. Relative water content (RWC) showed strong associations with K and K/Na, emphasizing its role in maintaining ionic balance. This suggests that adequate water retention is crucial for sustaining potassium levels and minimizing sodium accumulation under salinity stress.

**Fig. 5:**
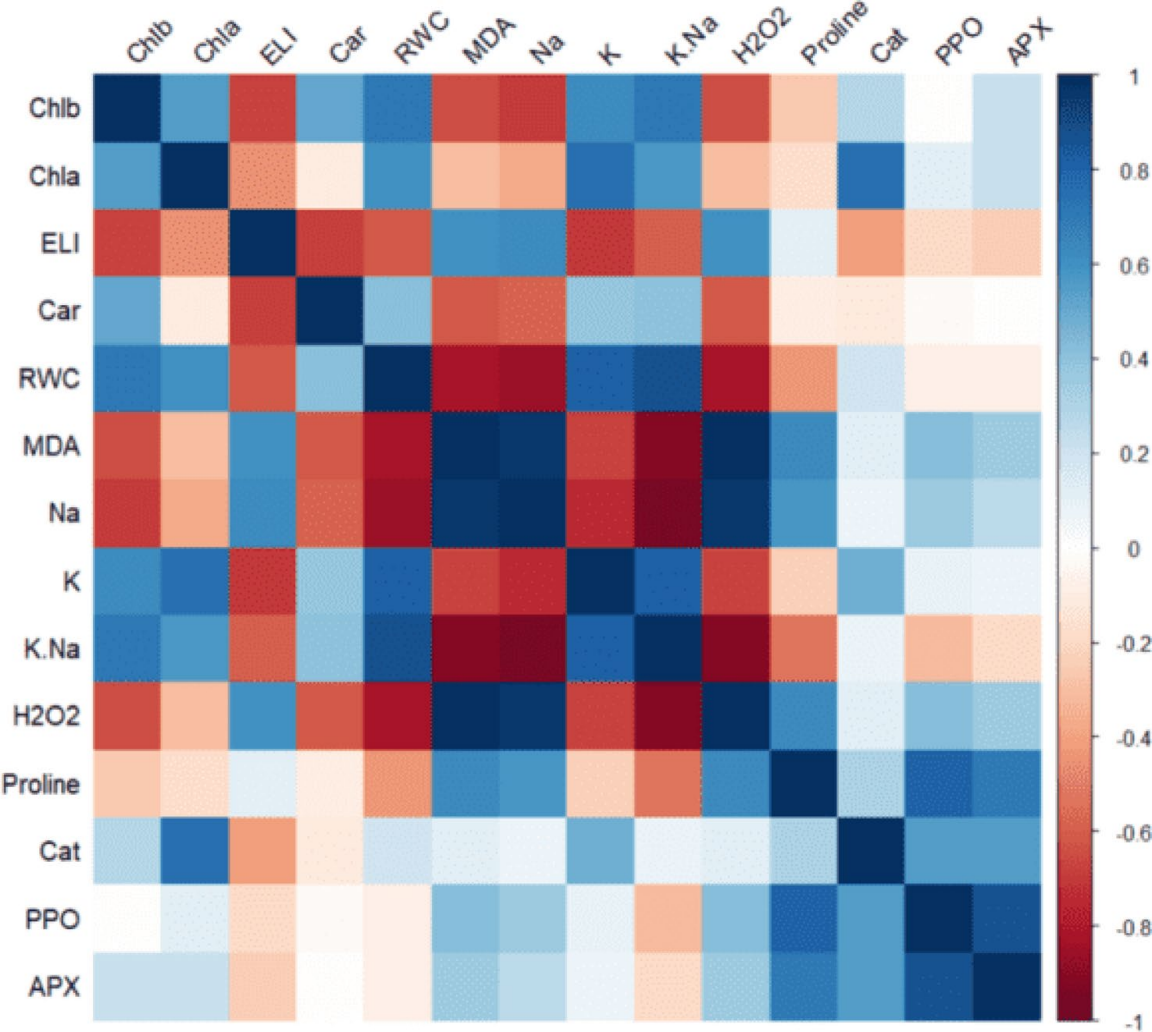
Significant correlations among physiological and biochemical factors, highlighting relationships between photosynthetic pigments, stress markers, lipid peroxidation (MDA), Hydrogen peroxide (H₂O₂), electrolyte leakage (ELI), and enzymatic activities (APX; Ascorbate peroxidase, CAT; Catalase, PPO; Polyphenol Oxidase).

Furthermore, MDA was closely linked to Na, H₂O₂, proline, PPO, and APX. This strong association highlights the interplay between oxidative damage markers and protective biochemical responses, demonstrating the importance of enzymatic defense mechanisms. Sodium (Na) exhibited significant relationships with H₂O₂ and proline, while potassium (K) correlated strongly with photosynthetic pigments, relative water content (RWC), and catalase activity. These findings suggest that ion homeostasis has a direct impact on both photosynthetic efficiency and antioxidant defense, thereby shaping the plant’s adaptation strategy. Notably, H₂O₂ was associated with oxidative stress markers, such as MDA and ELI. This correlation underscores the dual role of reactive oxygen species in both signaling and inducing cellular damage under stress conditions. Finally, proline showed a close relationship with PPO and APX, with these enzymatic activities exhibiting a strong mutual correlation (Fig. 5). The close association suggests that proline accumulation and antioxidant enzyme activity work in tandem to mitigate the effects of salinity-induced oxidative stress, enhancing overall plant resilience.

### Gene expression analysis

Salinity stress significantly influenced the relative expression of genes involved in ion transport and homeostasis, while Strigolactone hormone treatments further modified these effects. These findings highlight the dynamic interaction between environmental stressors and hormonal regulation, shaping plant adaptive responses at the molecular level.

**Fig. 6:**
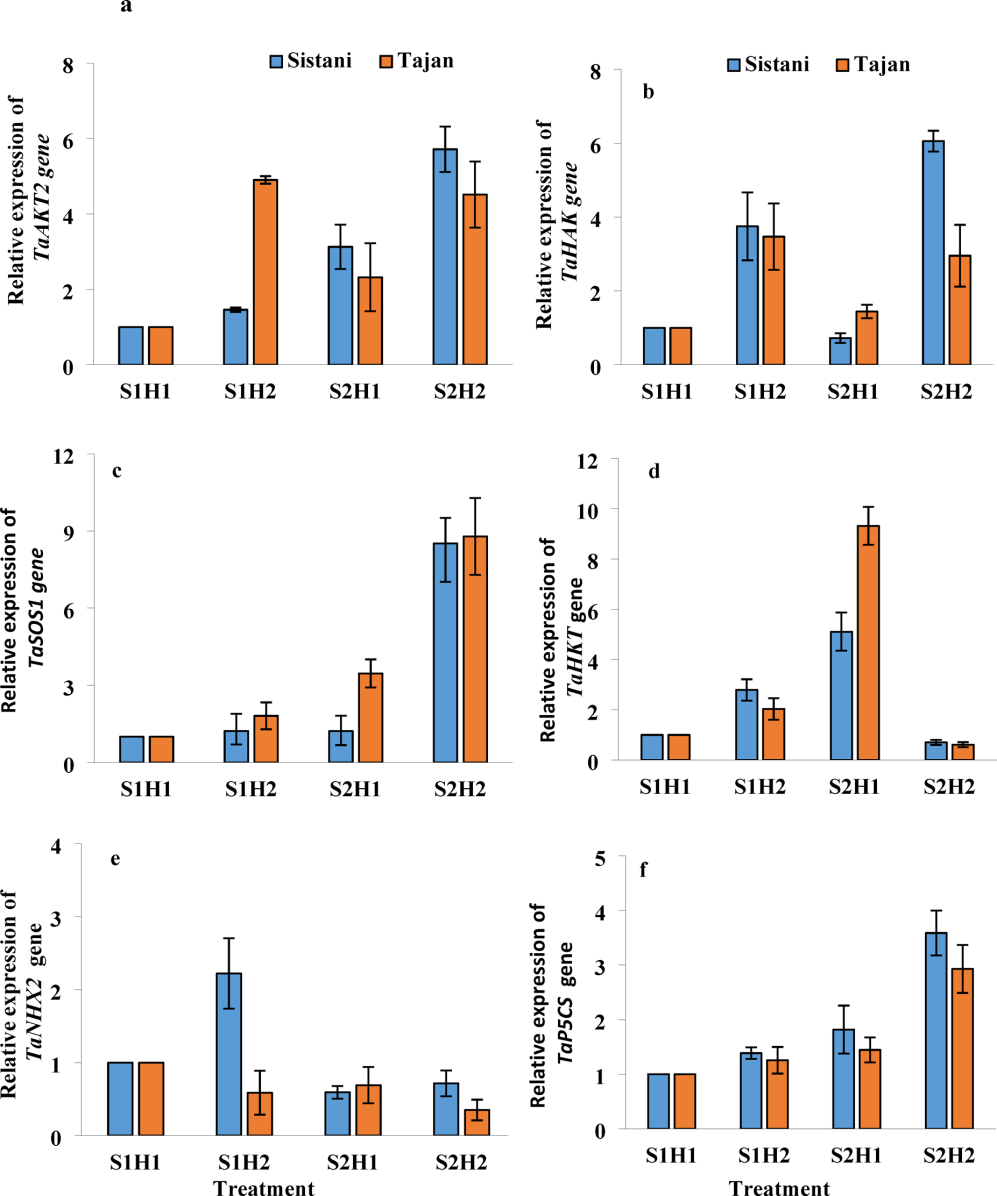
Effect of GR24 on Antioxidant and Ion Transport-Related Gene Expression in Wheat Cultivars (‘Tajan’ and ‘Sistani’) Under Salinity Stress. Treatments are as follows: S1: 0 dS/m NaCl, S2: 15 dS/m NaCl, H1: without hormone (GR24 at 0 µM), H2: with hormone (GR24 at 10 µM).

The highest expression of *TaAKT2* (Arabidopsis K + Transporter 2), an inward-rectifying potassium channel gene, was observed under S15 (15 dS/m salinity) and H2 (10 µM GR24) treatments for the Sistan variety, followed by the Tajan variety under similar conditions. This suggests that high salinity, combined with GR24 treatment, significantly enhances the expression of ion transport genes, thereby promoting cellular homeostasis under stress conditions (Fig. 6a).

In contrast, both varieties recorded significantly lower expression levels under S0 (no salinity) and H1 (0 µM GR24) treatments. This indicates that under non-stress conditions, baseline gene expression remains minimal, reinforcing the idea that these genes are primarily induced in response to external stressors.

Similarly, the expression of *TaHAK* (High-Affinity Potassium Transporter) peaked in the Sistan variety under S15 + H2, with the S0 + H2 treatment for the same variety showing the second-highest expression level. This highlights the essential role of potassium transport mechanisms in mitigating ionic imbalances under salinity stress (Fig. 6b).

The *TaSOS1* (Salt Overly Sensitive 1) gene, essential for sodium extrusion, exhibited the highest expression under S15 + H2 in the Tajan variety, followed by the Sistan variety. This suggests that GR24 treatment may enhance Na + efflux mechanisms, thereby reducing sodium toxicity and improving cellular integrity under high salinity conditions (Fig. 6c).

The expression of *TaHKT* (High-Affinity K+/Na + Transporter) was elevated in the Tajan variety under S15 + H1, highlighting its role in sodium regulation. This suggests that TaHKT serves as a crucial component in ion transport, enabling the selective removal of sodium even under moderate salinity conditions (Fig. 6d).

For *TaNHX2* (Sodium Hydrogen Exchanger 2), encoding a vacuolar Na+/H + antiporter, higher expression was observed in the Sistan variety compared to others, emphasizing its effectiveness in sodium compartmentalization. This suggests that vacuolar sequestration of sodium is a critical adaptation strategy in salt-resistant wheat genotypes (Fig. 6e).

Lastly, the expression of *TaP5CS* (Pyrroline-5-carboxylate Synthase), a pivotal enzyme in proline biosynthesis, was highest in the Sistan variety under S15 + H2 conditions, followed by the Tajan variety under the same conditions (Fig. 6f). This highlights the role of osmoprotectants, such as proline, in mitigating oxidative stress and maintaining cellular stability during exposure to high salinity levels. The heatmap clustering analysis revealed distinct gene expression patterns, indicating that specific genes clustered together based on their expression profiles. These clustering patterns provide valuable insights into the functional relationships between genes, suggesting potential co-regulation mechanisms in response to stress. Cluster 1 included *TaSOS1* and *TaP5CS*, Cluster 2 grouped *TaAKT2* and *TaHAK*, and Cluster 3 combined *TaHKT* and *TaNHX*. This grouping suggests that genes involved in ion transport, osmoprotectant synthesis, and sodium regulation may operate within coordinated molecular pathways. The clusters were further analyzed to show that the S15 and H2 treatments for both Sistan and Tajan varieties clustered together. This indicates that plants subjected to high salinity and hormone treatment exhibit similar gene expression profiles, reinforcing the role of GR24 in modulating stress-related gene activity. The S0 and H1 treatments for both Sistan and Tajan were grouped and subsequently joined with the S0 and H2 treatments for Sistan. These results suggest that low salinity and minimal hormone treatment lead to similar gene expression patterns, reflecting the absence of significant stress-induced transcriptional changes. Additionally, the S15 and H1 treatments for both Sistan and Tajan clustered together and were joined with the S0 and H2 treatments for Tajan. This implies that certain treatment combinations exhibit overlapping regulatory effects, potentially influencing shared molecular pathways despite differing levels of salinity exposure.

**Fig. 7:**
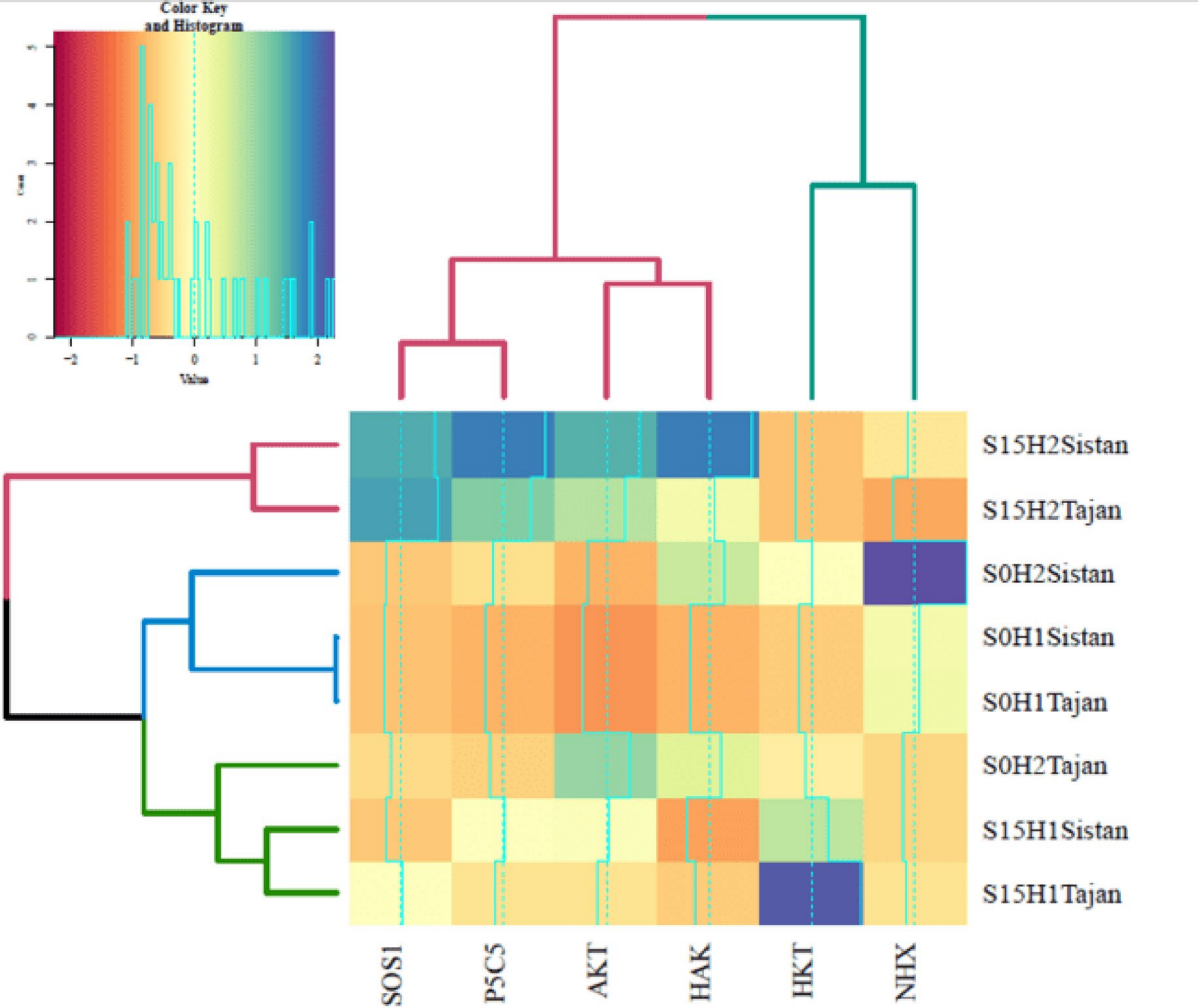
Heatmap Clustering Analysis of Gene Expression in Wheat Cultivars Under Salinity Stress and GR24 Treatments. The heatmap illustrates the clustering of ion transport and antioxidant-related gene expression in sensitive (Tajan) and tolerant (Sistani) wheat cultivars under salinity stress levels (0, 15 dS/m NaCl) and GR24 foliar treatments (H1: 0 µM and H2: 10 µM). Differential expression patterns are represented by color intensity, revealing treatment-specific variations in gene activity.

This clustering reveals the impact of salinity and hormonal treatments on gene expression patterns, underscoring the complexity of gene regulation in response to environmental stress (Fig. 7). Such insights lay the groundwork for future studies examining the genetic mechanisms underlying salinity tolerance in wheat.

### Correlation analysis

The correlation analysis revealed significant relationships among the genes studied, highlighting the intricate interplay of gene expression in response to salinity and hormonal treatments. These correlations provide insights into the potential genetic pathways involved in stress adaptation, revealing interactions that may be crucial for improving salinity tolerance.

A strong positive correlation was observed between *TaSOS1* and *TaP5CS*, indicating co-expression under similar conditions, while *TaHAK* and *TaAKT2* also exhibited a high correlation, suggesting possible co-regulation or functional linkage. Such co-expression patterns indicate that these genes may work in synergy, coordinating multiple physiological processes to enhance plant resilience. Furthermore, *TaAKT2* showed significant positive correlations with both *TaSOS1* and *TaP5CS*, reinforcing its central role in the stress response network. This central positioning suggests that *TaAKT2* acts as a key modulator in ion transport and osmotic stress regulation, facilitating broader stress-response mechanisms.

**Fig. 8:**
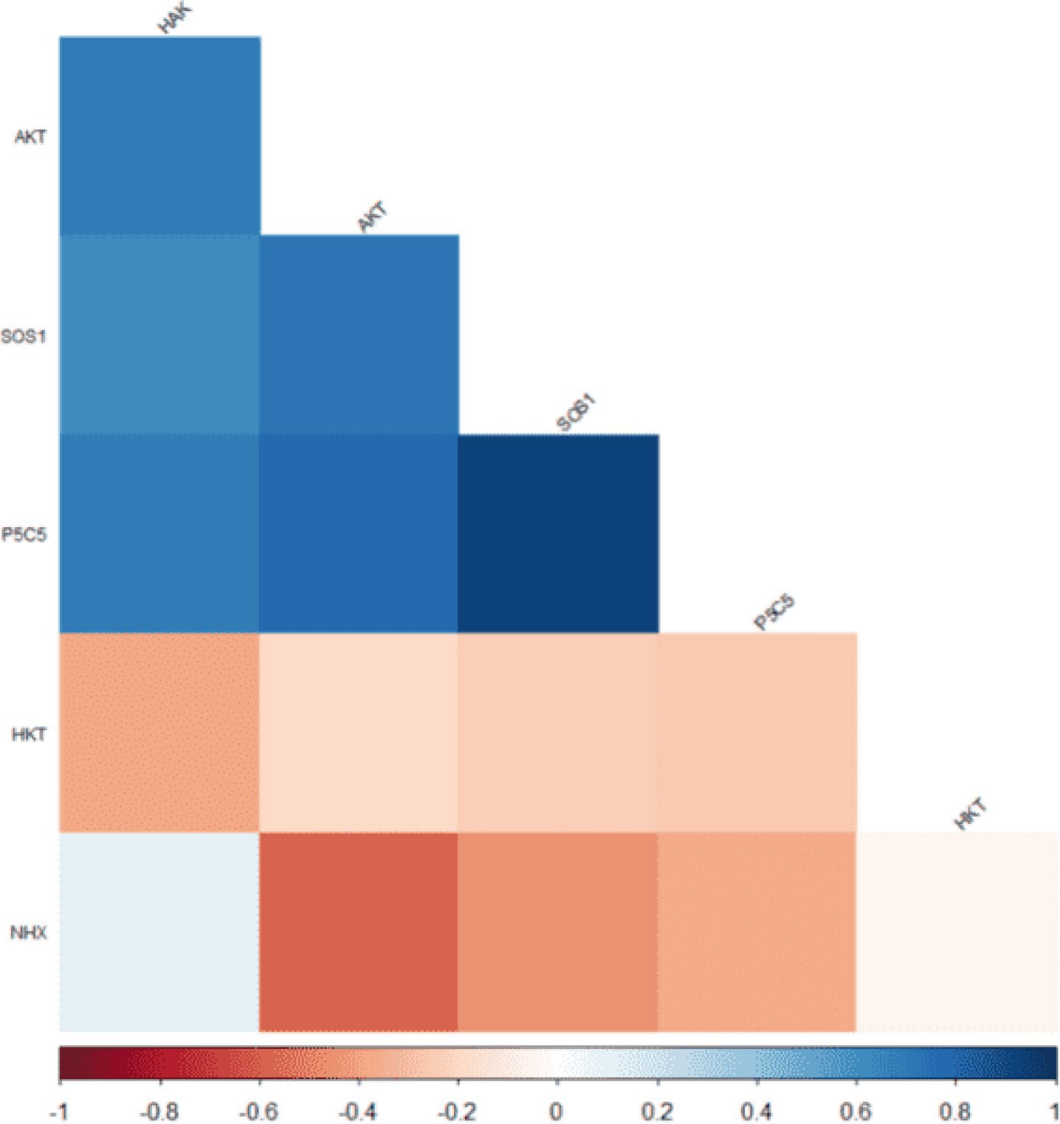
Correlation Analysis of Ion Transport and Antioxidant-Related Gene Expression in Wheat Cultivars Under Salinity Stress and GR24 Hormone Treatments. This figure displays the correlation analysis of ion transport and antioxidant-related gene expression in Tajan (sensitive) and Sistani (tolerant) wheat cultivars exposed to salinity stress levels (0, 5, and 15 dS/m NaCl) and GR24 foliar treatments (0 µM and 10 µM).

However, very low correlations were found between *TaNHX2* and both *TaHKT* and *TaSOS1*, as well as between *TaNHX2* and *TaP5CS*, indicating distinct regulatory mechanisms. These weak associations imply that *TaNHX2* may function independently or be regulated by different signaling pathways under salinity stress. Additionally, *TaHKT* and *TaHAK* exhibited a low correlation, suggesting they are not co-regulated or functionally connected under the same conditions. This lack of co-expression indicates that while both genes contribute to ionic balance, they may operate through separate mechanisms or in response to different environmental stimuli. This analysis highlights the complexity of gene regulatory networks and the specific roles of these genes in tolerance through their differential expression patterns (Fig. 8). Understanding these interactions can support future research aimed at manipulating gene expression to enhance crop resilience.

### PCA analysis of salinity response in Sistan and Tajan genotypes

The principal component analysis (PCA) provides a comprehensive overview of the critical traits governing salinity response in the Sistan and Tajan genotypes, highlighting distinct patterns of resilience and sensitivity. This approach helps visualize complex relationships among traits, offering more profound insight into genotype-specific adaptations.

For the resistant “Sistan” genotype, the first principal component (Dim1) explains 52.1% of the total variance. Key contributors include *HAK*, Cat, SOS1, APX, PPO, Car, *AKT*, Proline, and Chla, representing essential biochemical and physiological traits. The substantial contributions of these traits indicate the genotype’s ability to activate multiple defense mechanisms simultaneously, reinforcing its salinity resistance.

The second principal component (Dim2), accounting for 33.2% of the variance, underscores complementary adaptive traits, including K, Chlb, and RWC. These traits contribute to water retention, ion balance, and chlorophyll content, further enhancing the genotype’s overall tolerance to salinity stress. The combination of Dim1 and Dim2 captures the multifaceted nature of salinity adaptation, reflecting both primary and secondary defense strategies.

**Fig. 9:**
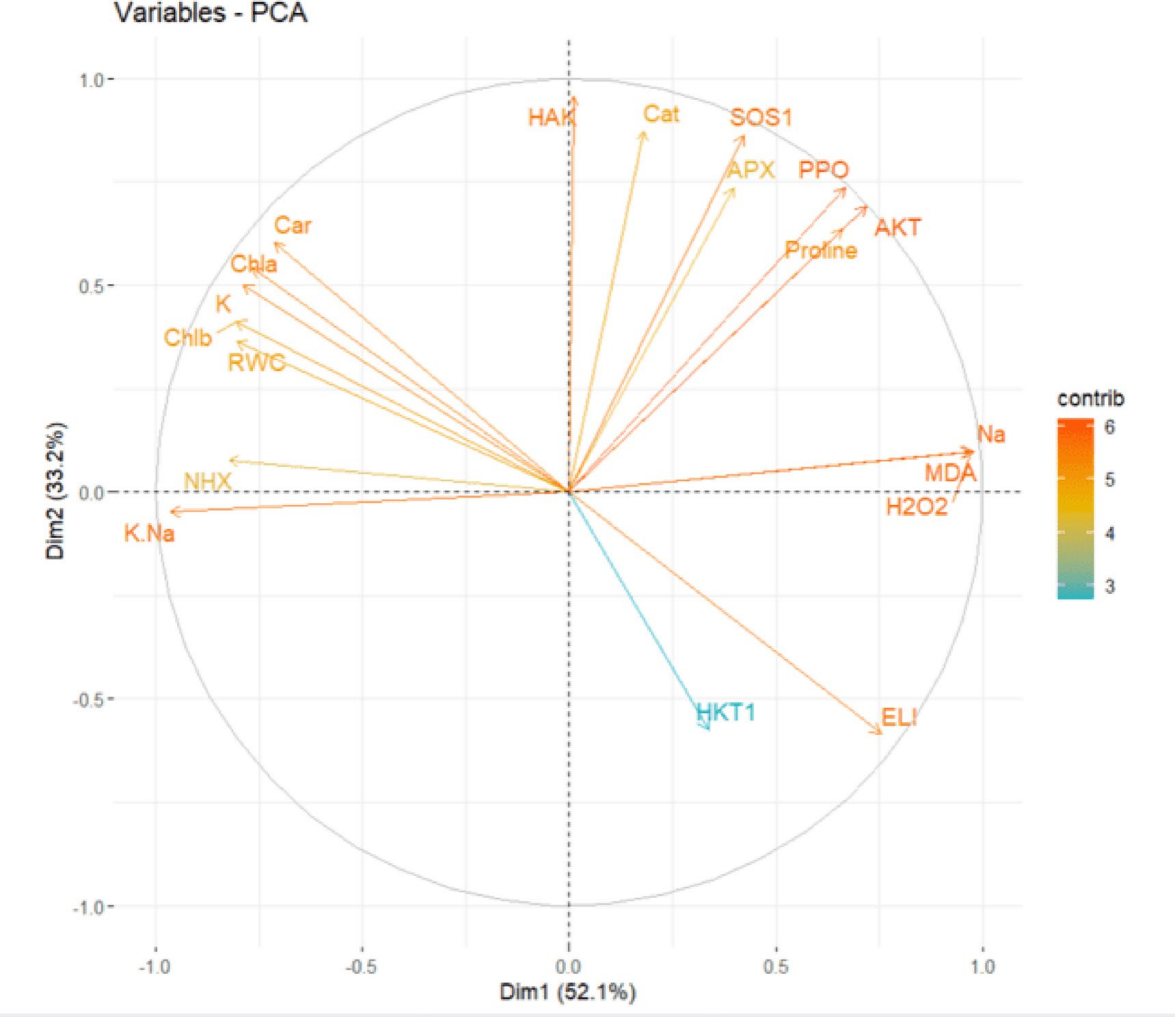
PCA analysis of the Sistan genotype, which is resistant to salinity. Dim1 (51%) highlights contributions from variables such as SOST, Cat, and PPO, while Dim2 (36.7%) is associated with variables like Na and *NHX*.

Notably, variables such as *HKT1*, ELI, Na, and NHX show negative contributions, suggesting potential antagonistic effects on the resistance profile of the Sistan genotype (Fig. 9). Understanding these negative influences can guide efforts to minimize their impact through targeted breeding or molecular interventions.

In contrast, the sensitive “Tajan” genotype exhibits a slightly different trait distribution. Dim1 explains 51% of the variance, with significant contributions from SOS1, APX, *AKT*, Cat, PPO, and Carotenoid. This suggests a heavy reliance on metabolic and biochemical processes for coping with salinity stress rather than structural or osmotic adjustments.

Dim2, explaining 36.7% of the variance, highlights traits such as Chla, Chlb, and RWC, which are critical for water regulation and photosynthetic processes. These attributes indicate that photosynthetic efficiency plays a significant role in maintaining physiological stability despite salt-induced oxidative stress.

While Dim1 and Dim2 collectively capture 87.7% of the dataset’s variability, negative contributions from variables such as Na, *NHX*, and *HKT1* indicate potential detrimental impacts on salinity tolerance. The relatively high influence of these stress-related ions suggests a weaker ion regulation mechanism compared to the more resistant Sistan genotype.

Clustering traits like SOS1 and PPO in the PCA biplot suggests interconnected biochemical roles. These relationships highlight how multiple stress-response pathways function synergistically to enhance overall survival under salinity stress.

**Fig. 10:**
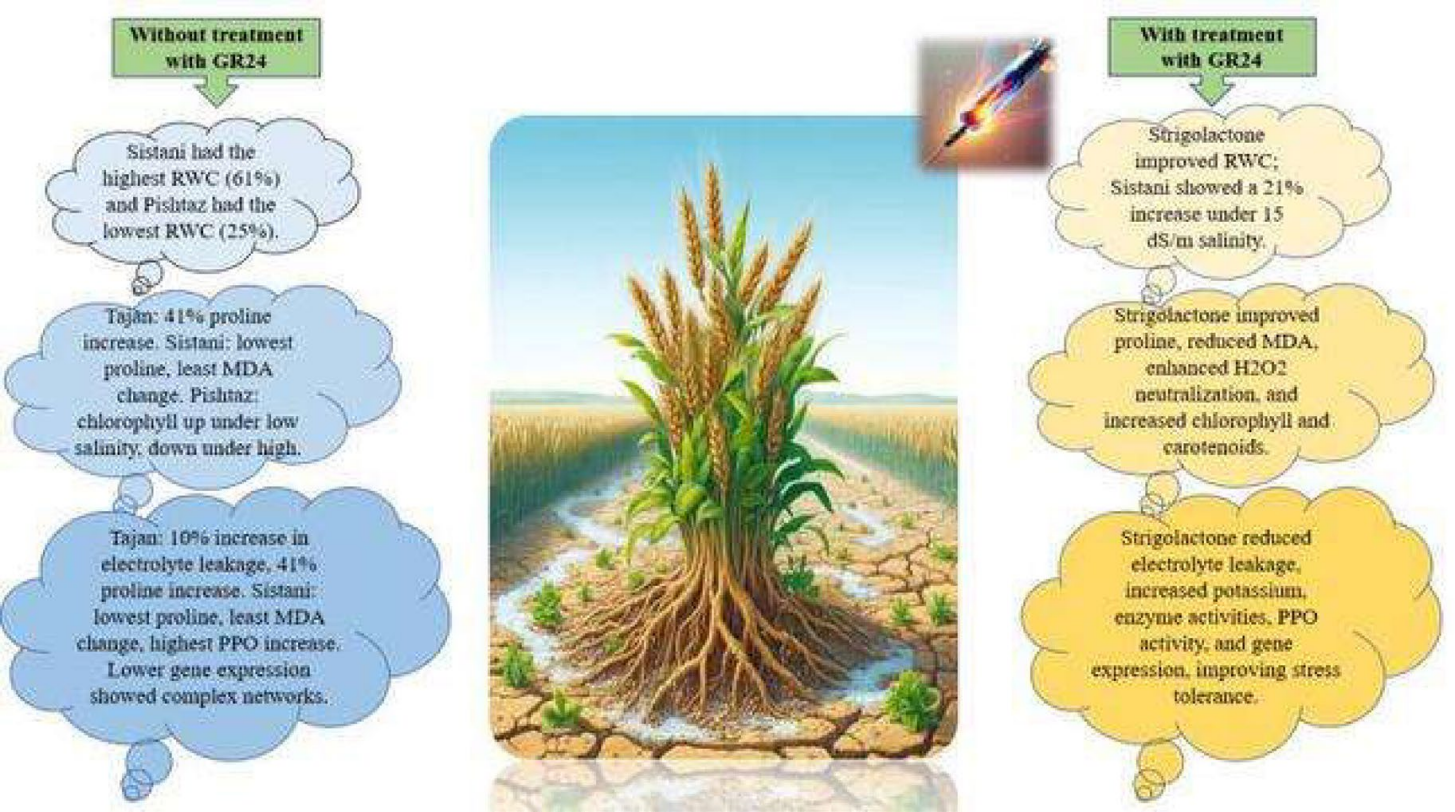
The effects of GR24 treatment on RWC, proline, MDA, ELI, chlorophyll, and enzymatic activities in wheat varieties under saline conditions.

In contrast, opposing traits (e.g., Na versus RWC) reveal trade-offs in adaptive strategies (Fig. 10). This balance between ion transport and water retention reflects the genotype’s prioritization of specific defense mechanisms over others in response to salinity. When comparing the Sistan and Tajan genotypes, key similarities and differences emerge. Both genotypes highlight the importance of antioxidant enzymes (e.g., APX, CAT) and osmoprotectants (e.g., Proline, RWC) in driving the salinity response. These common traits suggest a universal defense strategy among wheat genotypes, albeit with variations in efficiency. However, the Sistan genotype strongly emphasizes photosynthetic pigments (Chla, Chlb) and ion transport regulation (e.g., K, Na), suggesting a more balanced strategy combining metabolic efficiency and water-ion homeostasis. The ability to maintain chlorophyll integrity under stress contributes significantly to its superior resilience.

In contrast, the Tajan genotype relies heavily on specific metabolic traits, highlighting its limited adaptability under salinity stress. This reliance indicates a more reactive rather than proactive stress response, potentially limiting its long-term viability in saline environments.

Overall, the PCA analysis provides valuable insights into the resilience mechanisms of the Sistan genotype and the vulnerabilities of the Tajan genotype. These findings provide a solid framework for targeted breeding programs and strategies to improve stress resilience in crops exposed to salinity challenges. The integration of PCA in plant breeding further enhances the ability to predict genotype performance under varying environmental conditions.

Figure 10 PCA analysis of the Tajan genotype, sensitive to salinity. Dim1 (51%) reflects contributions from variables such as SOST, Cat, and PPO, while Dim2 (36.7%) aligns with Na and *NHX*.

## Discussion

Salinity stress severely impacts plant physiological functions, primarily through osmotic imbalance, ion toxicity, and oxidative damage^10,39–43^. GR24, a synthetic Strigolactone analog, has emerged as a key biochemical regulator that enhances plant resilience by maintaining osmotic balance, reinforcing antioxidant defenses, stabilizing membrane integrity, and modulating ion homeostasis^44–47^. Its exogenous application has been demonstrated to improve RWC, chlorophyll stability, and overall photosynthetic efficiency, solidifying its role as an adaptive stress modulator^48,49^.

Maintaining RWC is essential for plant survival under abiotic stresses such as drought and salinity^50–53^. The application of GR24 has been shown to optimize cellular water transport by regulating aquaporin activity, modulating stomatal dynamics to balance transpiration and water conservation, and interacting with ABA signaling pathways to mitigate dehydration risk ^(46–49, 56, 33)^. Experimental findings confirm that GR24 treatment significantly improves relative water content (RWC) in sunflower plants, reinforcing its role in osmotic regulation and ensuring physiological stability under saline conditions.

Oxidative damage induced by excessive accumulation of ROS is a significant consequence of salinity stress^54,55^. GR24 contributes to oxidative homeostasis by upregulating antioxidant enzymes, including SOD, CAT, and APX. It effectively reduces lipid peroxidation markers, including MDA and H₂O₂, thereby preserving membrane integrity and mitigating oxidative disruptions^56–58^. Additionally, GR24 enhances chlorophyll retention, preventing ROS-triggered degradation, and supports sustained photosynthetic activity under stress conditions.

Salinity-induced ionic imbalance, primarily driven by Na⁺ and Cl⁻ toxicity, disrupts intracellular homeostasis and interferes with essential metabolic processes^59,60^. GR24 facilitates ionic equilibrium by regulating K⁺ and Ca²⁺ channels, mitigating ethylene-mediated chlorophyll degradation, and influencing auxin transport to enable adaptive physiological responses under stress conditions^61–63^. One of its critical functions in salinity tolerance is the promotion of sodium exclusion mechanisms, enhancing ionic stability and improving plant viability in affected tissues.

Beyond ion regulation, GR24 exerts broad control over stress-responsive gene networks^33^. It strengthens enzymatic defense mechanisms^64,65^reinforces ROS detoxification pathways^66,67^and optimizes Photosystem II efficiency to ensure consistent photosynthetic performance despite environmental stressors^68,69^. Furthermore, GR24 enhances secondary metabolite biosynthesis^70,71^increasing the accumulation of stress-protective compounds that contribute to cellular resilience. Its involvement in ABA, ethylene, and auxin signaling highlights its importance in hormonal crosstalk, orchestrating plant adaptation under adverse conditions^72–74^.

Ion leakage, a widely recognized indicator of membrane integrity under environmental stress, is significantly reduced by GR24 application^75,76^. It reinforces membrane stability by decreasing electrolyte leakage, enhancing lipid membrane resilience, and supporting cytoprotective mechanisms that alleviate oxidative damage. The destabilization of membranes due to salt stress is particularly pronounced in salt-sensitive plant varieties; however, GR24 treatment has been shown to reduce ion leakage while maintaining stomatal function, relative water content (RWC), and overall chlorophyll stability, ultimately contributing to increased stress tolerance.

Lipid peroxidation, quantified through malondialdehyde (MDA) accumulation, serves as an indicator of oxidative membrane damage. Elevated MDA levels under stress conditions result in protein cross-linking and DNA degradation, leading to cellular deterioration^77,78^. Research has shown that GR24 application significantly reduces MDA accumulation and enhances the activity of antioxidant enzymes such as SOD and POD, reinforcing plant tolerance against salt-induced oxidative stress^79,80^. Comparisons between salt-sensitive and salt-tolerant genotypes reveal lower MDA levels and higher antioxidant activity in plants treated with GR24, further supporting its role as a stress modulator.

The intricate hormonal interplay between GR24, ABA, and H₂O₂ contributes significantly to salinity stress adaptation^81–83^. Studies indicate that ABA regulates Strigolactone biosynthesis via H₂O₂ signaling, highlighting its potential in improving salt tolerance^33^. Additionally, GR24 independently modulates stomatal responses and oxidative defense systems, thereby solidifying its role in stress mitigation. Maintaining a balanced cytosolic K⁺/Na⁺ ratio is crucial for cellular stability under saline conditions, highlighting ion regulation as a vital determinant of stress resilience.

These physiological and biochemical insights into GR24’s role in alleviating salinity stress lay the foundation for future advancements in sustainable agriculture^47,84^. Further investigations into its molecular interactions, particularly its influence on ABA, ethylene, and gibberellin pathways, will enhance our understanding of its regulatory mechanisms and broaden its application in crop improvement strategies. Expanding research on GR24-mediated stress responses across diverse plant species and environmental conditions will facilitate the development of optimized agricultural practices, thereby improving stress resilience in crops cultivated under saline conditions.

Ultimately, integrating GR24-based approaches into modern agricultural systems offers promising avenues for enhancing plant tolerance against environmental stressors. The multifunctionality of GR24 in osmotic regulation, antioxidant defense activation, ion homeostasis, and hormonal modulation positions it as a valuable tool for advancing crop resilience in saline environments, ensuring long-term agricultural sustainability and productivity.

Genetic and physiological differences among plant species and varieties influence their response to stress, highlighting the potential for breeding and genetic engineering to develop stress-tolerant crops, ensuring food security under changing climatic conditions (Fig. 10).

## Materials and methods

### Plant materials and growth conditions

Seeds of three wheat cultivars (Sistan, Tajen, and Pishtaz) were obtained from the Gene Bank of the College of Agriculture and Natural Resources, University of Tehran, Iran. The seeds were surface-sterilized using 10% sodium hypochlorite for 1 min and soaked in distilled water for 24 h. Five seeds per cultivar were sown in 7-liter pots containing a soil mixture composed of field soil, leaf mold, and perlite (2:1:1 ratio). The pots were maintained in a growth chamber under controlled conditions: 16-hour light/8-hour dark photoperiod, day/night temperatures of 24 °C ± 2 °C and 20 °C ± 2 °C, respectively, and 60% ± 5% relative humidity.

### Experimental design and treatments

The experiment followed a factorial design within a completely randomized framework, with **three replications. Salinity stress was applied at three levels (0, 5, and 15 dS/m), and the synthetic strigolactone GR24 was used at two concentrations (0 and 10 µM). Salinity levels were adjusted by controlling the electrical conductivity of the soil solution and gradually increasing NaCl concentrations in the irrigation water. Plants were irrigated with tap water until the 3–4 leaf stage, after which salinity stress was induced. GR24 was administered as a foliar spray at the tillering stage, applied over two consecutive days. Control plants received an equivalent volume of distilled water.

### Sample collection

One week after the final GR24 treatment, aerial parts of the plants were harvested, wrapped in aluminum foil, and immediately transferred to the laboratory for storage in liquid nitrogen. Samples were maintained at – 80 °C for subsequent analyses.

### Relative water content (RWC) measurement

To determine the relative water content (RWC) of leaves, a fully expanded leaf was excised and weighed to record the fresh weight (FW). The leaf was then immersed in distilled water at room temperature for 24 h to reach full turgidity. After removing excess surface water, the turgid weight (TW) was recorded. The leaf was subsequently oven-dried at 70 °C for 72 h to determine the dry weight (DW). RWC was calculated using the formula:^85–88^:

$$\:RWC=\frac{\text{F}\text{W}-\text{D}\text{W}\:}{\text{T}\text{W}-\:\text{D}\text{W}}\times\:100$$

### Electrolyte leakage index (ELI) measurement

A fully expanded leaf was excised and placed in a Falcon tube containing 15 mL of distilled water. Samples were incubated on a shaker at 25 °C for 24 h to facilitate ion leakage. The initial electrical conductivity (EC₁) was measured using an EC meter. The tubes were then subjected to a boiling water bath (95 °C) for 1 h, and the final electrical conductivity (EC₂) was recorded. The ELI was determined using the formula:^89^

$$\:ELI=\frac{EC1}{EC2}\times\:100$$

### Sodium (Na^+^) and potassium (K^+^) content measurement

Plant tissue was ashed to determine the concentrations of Na⁺ and K⁺ ions. Approximately 0.5 g of dried leaf tissue (dried at 70 °C for 48 h) was finely ground and ashed in an electric furnace at 550 °C for 5 h. The ash was dissolved in 5 mL of 2 N HCl, then diluted to 50 mL with distilled water after 15–20 min. The solution was filtered through Whatman filter paper, and the concentrations of Na⁺ and K⁺ were measured using a flame photometer (JENWAY)^90^.

### Leaf sample preparation

Prior to physiological and biochemical analyses, leaf samples were ground in liquid nitrogen using an autoclaved mortar and pestle^91^. The powdered samples were stored in autoclaved containers at – 80 °C until further use.

### Antioxidant enzyme activity measurement

#### Enzyme extract preparation

All equipment was autoclaved twice to ensure sterility before preparing the extraction buffer. For total protein content and enzyme activity measurements, 2 mL tubes containing 0.1 g of powdered leaf sample were used. Each tube was filled with 1 mL of cold phosphate buffer (pH 7.6), prepared by dissolving 8 g of NaCl, 0.2 g of KCl, 1.44 g of Na₂HPO₄, and 0.24 g of KH₂PO₄ in distilled water to a final volume of 1 L^92,93^. The buffer contained 0.01 mM EDTA and 1% PVP, prepared the day before and stored at 4 °C. Samples were vortexed for 20 s, incubated at 4 °C for 30 min, and then centrifuged at 15,000 g for 20 min at 4 °C. The supernatant was collected, stored in pre-cooled tubes in liquid nitrogen, and kept at – 20 °C* until use^94^.

#### Total protein content measurement

##### Bradford reagent preparation

To prepare the Bradford reagent, 0.1 g Coomassie Brilliant Blue G-250 was dissolved in 50 mL of 96% ethanol, followed by the addition of distilled water in an Erlenmeyer flask. The solution was stirred until the dye was completely dissolved. Additional distilled water was added, followed by the gradual addition of 100 mL of orthophosphoric acid. The final solution was refrigerated for several hours, diluted to a 1 L final volume, filtered through two layers of filter paper, and stored at 4 °C wrapped in foil^95^.

##### Standard stock solution and standard curve Preparation

A standard stock solution was prepared by dissolving 50 mg of bovine serum albumin (BSA) in 50 mL of distilled water. Serial dilutions were performed to obtain concentrations ranging from 0 to 1000 µg/mL. The absorbance of these standards was measured, and a standard curve was constructed.

##### Sample protein concentration measurement

For protein concentration measurement, 200 µL of Bradford reagent was added to each well of a 96-well plate, followed by 10 µL of enzyme extract. The mixture was gently mixed using a pipette and incubated for 20 min. Absorbance was read at 595 nm^96^.

#### Catalase (CAT) activity

To determine catalase activity, 0.6 g of cobalt nitrate (Co(NO₃)₂·6 H₂O) was dissolved in 30 mL of distilled water, 2.7 g of sodium bicarbonate (NaHCO₃) in 30 mL of distilled water, and 0.3 g of sodium hexametaphosphate (NaPO₃)₆ in 30 mL of distilled water. From these solutions, 3 mL of cobalt nitrate, sodium hexametaphosphate, and 27 mL of sodium bicarbonate were mixed sequentially. Separately, 300 µL of 30% H₂O₂ was diluted with phosphate buffer to a final volume of 30 mL, yielding a 10 mM H₂O₂ solution.

For the assay, 20 µL of enzyme extract was transferred into wells of a 96-well plate using a multi-channel pipette, followed by the addition of 40 µL of phosphate buffer and H₂O₂ solution. The mixture was incubated for 2 min. Afterward, 240 µL of the combined sodium bicarbonate, sodium hexametaphosphate, and cobalt nitrate solution was added, and the mixture was incubated for 10 min. Absorbance was measured at 440 nm^97^.

Catalase activity was calculated using the formula:

$$\:\:\:\text{k}\text{U}=\frac{2.303}{\text{t}}\:\text{l}\text{o}\text{g}\:\frac{\text{s}^\circ\:}{\text{s}}$$

where (t) is the time (10 min), (s°) is the absorbance of the control, and (s) is the absorbance of the sample.

#### Ascorbate peroxidase (APX) activity

APX activity was assessed by preparing a reaction mixture containing 1984 µL of 50 mM phosphate buffer, 4 µL of 100 mM ascorbic acid, and 10 µL of enzyme extract in a cuvette. Subsequently, 4 µL of 30% H₂O₂ was added using a pipette, and the mixture was gently mixed. The absorbance was measured at 290 nm for 5 min at 25-second intervals^98^ .

APX activity was calculated as follows:

$${\text{Activity(U/ml) = change}}\,{\text{of}}\,{\text{absorbance(}}\Delta {\text{A470)/min/ml}}\,{\text{of}}\,{\text{the}}\,{\text{sample}}$$

#### Guaiacol peroxidase (GPOX) activity

A 96-well microplate enzymatic assay was performed to quantify GPOX activity. 20 µL of enzyme extract was dispensed into each well, followed by 200 µL of 100 mM phosphate buffer (pH 7.0) and 20 µL of 200 mM guaiacol solution. The reaction mixture was gently mixed to ensure homogeneity. Subsequently, 32 µL of 30% H₂O₂ was added to initiate the reaction, carried out under dark conditions to prevent H₂O₂ degradation. Absorbance was monitored at 470 nm using a microplate reader, with readings taken at 40-second intervals over 4 min^99^. The oxidation of guaiacol to tetra-guaiacol indicated GPOX activity, quantified by the rate of absorbance change.

GPOX activity was calculated as follows:

$${\text{Activity(U/ml) = change}}\,{\text{of}}\,{\text{absorbance(}}\Delta {\text{A470)/min/ml}}\,{\text{of}}\,{\text{the}}\,{\text{sample}}$$

#### Polyphenol oxidase (PPO) activity

For PPO activity, 10 µL of enzyme extract was added to a 96-well plate, followed by 300 µL of phosphate buffer and 5 µL of 100 mM pyrogallol solution. The mixture was gently mixed using a multi-channel pipette. Absorbance was measured at 420 nm over 4 min, with readings taken at 40-second intervals. One unit of PPO activity was defined as the amount of enzyme converting pyrogallol to purpurogallin per minute^100^.

PPO activity was calculated as follows:

$${\text{Activity(U/ml) = change}}\,{\text{of}}\,{\text{absorbance(}}\Delta {\text{A420)/min/ml}}\,{\text{of}}\,{\text{the}}\,{\text{sample}}$$

#### Lipid peroxidation (MDA) measurement

To measure lipid peroxidation, 0.1 g of powdered sample was incubated with 2 mL of 10% trichloroacetic acid (TCA) and centrifuged at 12,000 g for 10 min at 4 °C. 400 µL of the supernatant was transferred to a new tube and mixed with 1600 µL of 20% TCA containing 0.5% thiobarbituric acid (TBA). The mixture was vortexed and incubated at 95 °C for 1 h in a hot water bath, followed by cooling on ice for 20 min.

The samples were centrifuged at 12,000 g for 10 min, and 300 µL of the supernatant was transferred to a 96-well plate for absorbance measurement at 532 nm and 600 nm The MDA content was calculated using the formula^101^:

$${\text{MDA}}\,{\text{level}}\,{\text{(nmol/g FW) = }}\Delta {\text{(A532 - A600)/1}}{\text{.56 }}$$

#### Hydrogen peroxide (H₂O₂) measurement

To quantify H₂O₂ content, 0.1 g of leaf sample was ground in a mortar with 2 mL of 10% TCA. The extract was centrifuged at 10,000 rpm for 5 min. Subsequently, 250 µL of the supernatant was mixed with 250 µL of 100 mM phosphate buffer and 500 µL of 1 M potassium iodide (KI). Absorbance was measured at 390 nm using a spectrophotometer^102^.

#### Proline content measurement

For proline content measurement, 0.1 g of powdered sample was placed in a 2 mL tube, and 2 mL of 3% sulfosalicylic acid was added. Samples were vortexed and centrifuged at 15,000 g for 10 min at 4 °C. After centrifugation, 400 µL of the supernatantqwwwwwwwwwwwwwwww was transferred to a new tube.

Next, 400 µL of ninhydrin reagent (prepared by dissolving 1.25 g of ninhydrin in 30 mL of glacial acetic acid and adding 20 mL of 6 M phosphoric acid) and 400 µL of glacial acetic acid were added. The mixture was vortexed and incubated at 95 °C for 1 h in a hot water bath. The reaction was stopped by placing samples on ice for 20 min. Finally, 300 µL of the supernatant was transferred to a 96-well plate, and absorbance was measured at 520 nm using a plate reader.

$$\begin{aligned} & {\text{Proline}}\,{\text{concentration}}\,{\text{was}}\,{\text{calculated}}\,{\text{using}}\,{\text{a}}\,{\text{standard}}\,{\text{curve:}} \\ & {\text{Proline(}}{\upmu }{\text{mol proline/g FW) = (}}{\upmu }{\text{proline/ml)}} \div {\text{(115}}{\text{.5}}{\upmu }{\text{g/}}{\upmu }{\text{mol)}} \div {\text{(g sample/5)}} \\ \end{aligned}$$

#### Chlorophyll and carotenoid content measurement

To determine chlorophyll and carotenoid content, 0.05 g of powdered sample was mixed with 2 mL of 80% acetone and centrifuged at 12,000 g for 10 min at 4 °C. 300 µL of the supernatant was transferred to a 96-well plate, and absorbance was measured at 646, 663, and 470 nm using a spectrophotometer^103^.

$${\text{Chl a}} = \left( {{\text{12}}.{\text{25}} \times {\text{A663}}} \right) - \left( {{\text{2}}.{\text{79}} \times {\text{A646}}} \right)$$

$${\text{Chl b}} = \left( {{\text{21}}.{\text{5}} \times {\text{A646}}} \right) - \left( {{\text{5}}.{\text{1}} \times {\text{A663}}} \right)$$

$${\text{Total Chl}} = {\text{Chl a}} + {\text{Chl b}}$$

$${\text{Carotenoid}} = {\text{198}}\left( {{\text{1}}00 \times {\text{A47}}0} \right) - \left( {{\text{1}}.{\text{82}} \times {\text{Chl a}}} \right) - \left( {{\text{85}}.0{\text{2}} \times {\text{Chl b}}} \right)$$

### Total RNA extraction, cDNA synthesis, and gene expression analysis

#### RNA extraction and cDNA synthesis

Total RNA was extracted from 100 mg of powdered leaf samples using 1 ml of TRIzol reagent. The quality and quantity of the extracted RNA were assessed using a Nanodrop spectrophotometer (Thermo 1000 C) and 1% agarose gel electrophoresis^70,71,98,104,105^. The RNA samples were stored at -80 °C until further use. To eliminate genomic DNA contamination, the extracted RNA was treated with *DNase*. The first and second strands of cDNA were synthesized using the SMOBIO kit (Cat. No. RP1300) following the manufacturer’s instructions^106^.

#### Gene expression analysis

Gene expression in *T. aestivum* under salinity stress was analyzed using primers targeting stress-response-associated genes (*P5CS*,* NHX2*,* SOS1*,* HAK*,* AKT2*, and *HKT2*,*1*, as detailed in Table 1). Primer sequences were designed using Oligo software version 7 and validated for specificity using Primer BLAST (NCBI database). To ensure accurate normalization of gene expression levels, the *T. aestivum* Actin gene (GenBank accession number AB181991.1) was used as a housekeeping gene. This approach enhances reliability and consistency in qRT-PCR analyses.

#### Data analysis

Data were analyzed using SAS 9.4 (2021) and R software 4.3.2, employing analysis of variance (ANOVA) within a factorial design under a completely randomized framework. Mean comparisons were conducted using Duncan’s multiple range test at a significance level of 5%. Graphs were generated using Excel 2016. Gene expression data were analyzed using GraphPad Prism 6. Heatmap and correlation analyses were performed using R software to visualize and interpret the complex relationships and patterns within the data.

Primer sequences for genes associated with salinity stress response, including *P5CS* (Pyrroline-5-carboxylate Synthase), *NHX2* (Wheat *NHX* Antiporter Gene), *SOS1* (Salt Overly Sensitive 1), *HAK* (High Affinity Potassium Transporter), *AKT2* (Arabidopsis K^+^ Transporter 2), and *HKT* (High Affinity K^+^/Na^+^ Transporter).

## Conclusions

The synthetic Strigolactone analog, GR24, emerges as a potent enhancer of salinity tolerance in wheat cultivars, demonstrating its ability to induce critical physiological and biochemical adaptations. A comprehensive analysis of plant responses to salt stress reveals that GR24 application increases proline content, acting as a key osmoprotectant that mitigates the harmful effects of salinity. The regulation of antioxidant enzyme activities, including APX, CAT, and PPO, underscores a sophisticated oxidative stress management mechanism that strengthens plant resilience.

Notably, GR24 treatment significantly improves leaf water retention, enhances chlorophyll and carotenoid levels, and optimizes potassium uptake while simultaneously reducing sodium accumulation, lipid peroxidation (as indicated by MDA levels), and H₂O₂ concentrations. These physiological changes contribute to superior cellular stability and stress endurance under saline conditions. A distinct reduction in GPX activity, coupled with an increase in PPO activity, underscores a nuanced balance within antioxidant defense pathways, providing deeper insights into Strigolactone-mediated stress responses.

Among tested wheat cultivars, Sistan and Tajan exhibit the most pronounced improvements, positioning GR24 as a promising candidate for enhancing salinity tolerance in wheat crops. These findings not only expand the understanding of Strigolactone-induced stress responses but also provide a foundation for advancing agricultural strategies aimed at increasing crop resilience in saline environments. The results pave the way for further molecular investigations and broader applications of GR24 in improving abiotic stress tolerance across diverse crop systems.

## Data Availability

The datasets generated and/or analyzed during the current study are available from the correspondingauthor upon reasonable request. No publicly accessible datasets were used or generated in this study. Allrelevant data supporting the findings are included within the manuscript.
